# Diagnostic accuracy of the Enferplex Bovine TB antibody test using individual milk samples from cattle

**DOI:** 10.1371/journal.pone.0301609

**Published:** 2024-04-30

**Authors:** Amanda O’Brien, Alastair Hayton, Keith Cutler, Andy Adler, Darren J. Shaw, John Clarke, Neil Watt, Gordon D. Harkiss

**Affiliations:** 1 Enfer Scientific, Naas, County Kildare, Ireland; 2 Synergy Farm Health, Maiden Newton, Dorset, United Kingdom; 3 Royal (Dick) School of Veterinary Studies & The Roslin Institute, University of Edinburgh, Edinburgh, United Kingdom; 4 MV Diagnostics Ltd, Roslin Innovation Centre, University of Edinburgh, Edinburgh, United Kingdom; Kafrelsheikh University Faculty of Veterinary Medicine, EGYPT

## Abstract

Bovine tuberculosis is usually diagnosed using tuberculin skin tests or at post-mortem. Recently, we have developed a serological test for bovine tuberculosis in cattle which shows a high degree of accuracy using serum samples. Here, we have assessed the performance of the test using individual bovine milk samples. The diagnostic specificity estimate using the high sensitivity setting of the test was 99.7% (95% CI: 99.2–99.9). This estimate was not altered significantly by tuberculin boosting. The relative sensitivity estimates of the test using the high sensitivity setting in milk samples from comparative skin test positive animals was 90.8% (95% CI: 87.1–93.6) with boosting. In animals with lesions, the relative sensitivity was 96.0% (95% CI: 89.6–98.7). Analysis of paired serum and milk samples from skin test positive animals showed correlation coefficients ranging from 0.756–0.955 for individual antigens used in the test. Kappa analysis indicated almost perfect agreement between serum and milk results, while McNemar marginal homogeneity analysis showed no statistically significant differences between the two media. The positive and negative likelihood ratio were 347.8 (95% CI: 112.3–1077.5) and 0.092 (95% CI: 0.07–0.13) respectively for boosted samples from skin test positive animals. The results show that the test has high sensitivity and specificity in individual milk samples and thus milk samples could be used for the diagnosis of bovine tuberculosis.

## 1. Introduction

Bovine tuberculosis (bTB) is a contagious disease caused mainly by *Mycobacterium bovis* (*M*. *bovis*) which affects cattle and other animals worldwide [1]. It causes major economic losses due to poor production performance and mandatory restrictions in trade and culling, with its associated losses, due to statutory control measures. Besides its economic effects, it is a major zoonosis in many countries owing to its tenacious nature and presence of resistance genes affecting different antitubercular medications [2]. The disease is difficult to control due in part to the presence of infected wildlife vectors and poor sensitivity of diagnostic tests, but also to lack of resources, and the unacceptability and affordability of test and cull control measures in many developing countries. The main diagnostic test used is the tuberculin test (TT), in which tuberculin is injected intradermally to cause a delayed-type hypersensitivity reaction in infected animals which can be detected and measured. Three different TTs are used: comparative cervical test (CCT) that measures the difference in increased skin thickness between purified protein derivatives from *M*. *bovis* (PPD-B) and *Mycobacterium avium* (PPD-A); single intradermal test (SIT) using PPD-B alone, either as the single cervical test (SCT) or the caudal (tail) fold test (CFT) [3]. The CCT diagnostic sensitivity (Dse) varies from 50% - 85%, while the diagnostic specificity (Dsp) ranges from 99.5% and 99.98% [4–6]. This variation has been ascribed to inherent poor sensitivity of the CCT and to performance errors during the application of the test resulting in reduced reliability [7]. The interferon gamma (IFNγ) test provides an increase in Dse (67% - 85.5%) though the Dsp is lower (range 85.0%– 99.6%) [4, 5], precluding its use as a screening test. The IFNγ test thus tends to be used in TT negative animals in bTB breakdown herds to detect infected animals missed by the TT.

However, despite the use of more stringent TT and IFNγ testing in control and eradication programmes, bTB still represents a major problem in many countries including the United Kingdom (UK) and Republic of Ireland (IE). The development of diagnostic tests based on other approaches could provide a way to detect *M*. *bovis* infected animals not detected by cell-mediated tests. Serology tests are used widely in veterinary infectious disease diagnosis and have potential in bTB diagnosis. Arguments against the use of antibody tests in bTB diagnosis include lack of sensitivity, variability and appearance of antibody only in the late stages of disease when animals become ‘anergic’ in the CCT and IFNγ test. However, this view is based on work published over 30 years ago using crude antigens and non-optimised tests [8, 9]. In the intervening years several antibody tests have been developed using recombinant antigens which show that antibody responses can be detected as early as 4 weeks after infection [10–13] and not just in the later stages. In addition, injection of tuberculin boosts antibody levels in samples taken 1–4 weeks post skin test [10, 14] thus increasing further the sensitivity of detection.

Recent work in humans, non-human primates and cattle has shown that use of multiple antigens increases the sensitivity of antibody tests further [15–19]. To take advantage of this information, we developed a multiplex serological test utilising 11 *M*. *bovis* antigens (Enferplex Bovine TB antibody test) which shows high sensitivity and specificity and detects infected cattle that are missed by TT and IFNγ tests [20]. In this study, we found serum antibodies in over 94% of CCT test positive animals which were clearly not ‘anergic’. In an early version of the Enferplex test, we detected serum antibodies from 5 weeks post experimental infection with *M*. *bovis* [21]. A recent study showed that the current WOAH validated version of the Enferplex test detected serum antibodies in 6/ 8 infected animals as early as 4 weeks post-infection [22]. In this experiment, 6/8 of these infected animals were positive in the IFNγ test at 4 weeks post-infection. The Enferplex bTB antibody test thus detects infection at all stages of the disease and not just in the late stages.

While obtaining serum for testing is straightforward in developed countries, the lack of trained veterinary personnel in many countries limits the use of serological tests. Milk samples, however, are much easier to obtain and could provide a medium that has more utility in resource poor settings. Many *in vitro* diagnostic tests for diseases in cattle and other livestock have been developed using milk as the test medium [23, 24]. For example, individual milk assays are available for pathogens such as *Bovine viral diarrhoea virus* (BVDV) [25], *Brucella abortus* (BA) [26], *Infectious bovine rhinotracheitis virus* (IBRV) [27], *Fasciola hepatica* (FH) [28, 29], *Neospora caninum* (NC) [30], and *Mycobacterium avium* subsp. *paratuberculosis* (Map) [31] in cattle.

In this report, we present data on the use of the Enferplex bTB antibody test using individual bovine milk as the test medium. The aims of the present study were to estimate the sensitivity and specificity of the Enferplex bTB antibody test using milk samples, and to perform a comparability study between individual milk and serum responses in cattle with and without bTB.

## 2. Materials and methods

### 2.1. Study design

**Fig 1:**
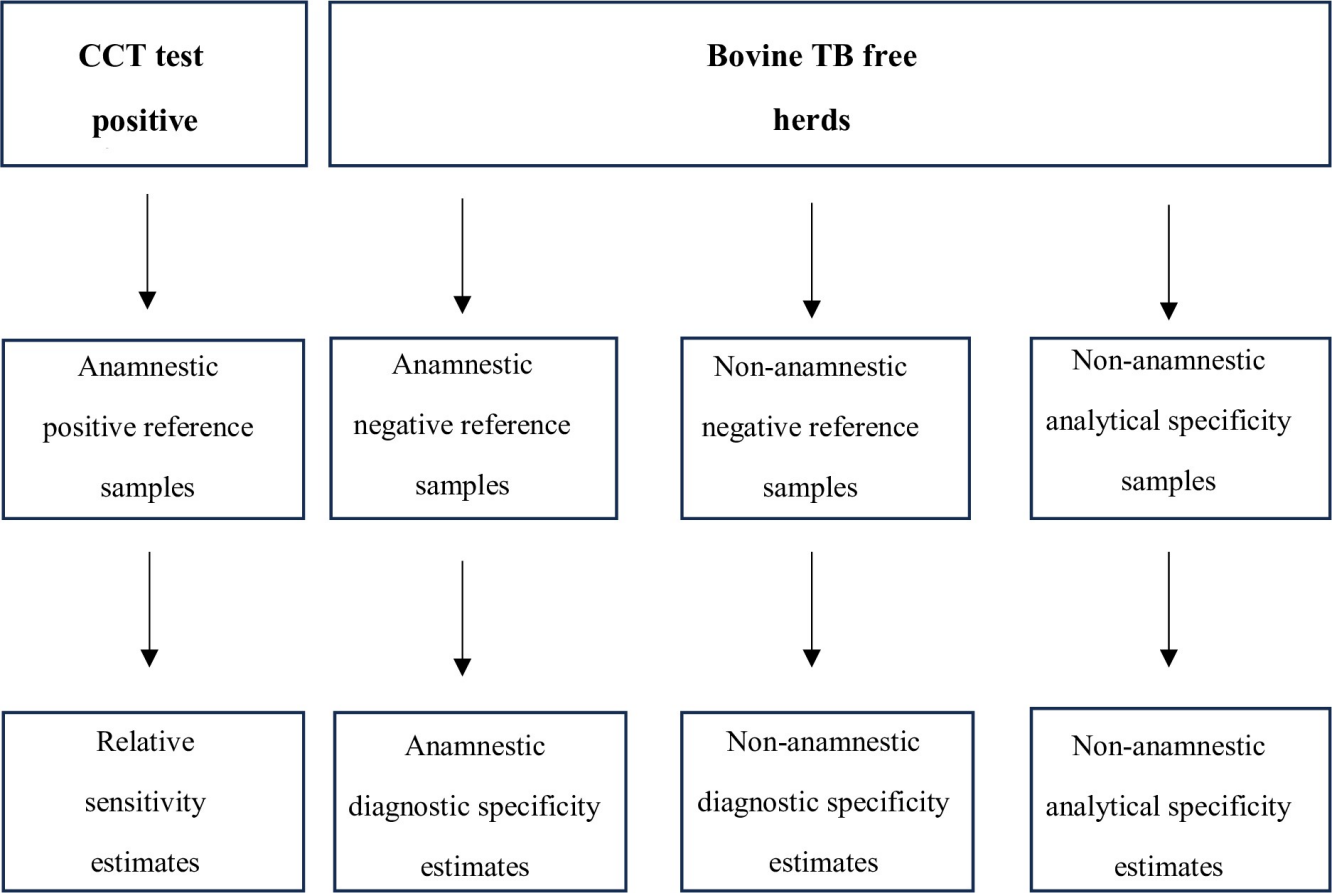
Project flow chart. Bovine milk sample were obtained from CCT positive bTB breakdown herds to obtain relative sensitivity estimates. Anamnestic and non-anamnestic milk samples were obtained from bTB free herds to determine diagnostic and analytical specificity estimates.

The study aimed to determine comparability between serum and milk samples from cattle for serodiagnosis of bTB using the Enferplex Bovine TB Antibody test, and to estimate the relative sensitivity (Rse) and Dsp of the test using milk samples (**Fig 1**). For this purpose, defined positive and negative reference individual milk samples from animals known to be infected with *M*. *bovis* or known to be from herds free of bTB respectively were tested. Test specificity was assessed further using samples from animals infected with *Map* and other common non-tuberculosis pathogens of cattle. The positive reference milk samples were obtained from bTB infected animals defined by results obtained in the routine statutory CCT carried out by the Animal and Plant Health Agency (APHA), UK. Milk and serum samples taken 5–30 days post CCT were regarded as being ‘boosted’ for antibody, while those taken outside this anamnestic window were regarded as being ‘non-boosted’ [20]. The negative reference milk samples were obtained from herds that were negative in the CCT in the low-risk area of England or from Scotland which holds Officially Tuberculosis Free (OTF) status and had no recent history of bTB and/or contact with animals likely to have been exposed to *M*. *bovis*. To determine the correlation between serum and milk, samples were obtained from CCT reactors during the anamnestic period. The positive and negative milk samples obtained encompassed wide variations in geographical location, cattle breed, age, husbandry, farm practices, and farm management within the UK.

### 2.2. Ethical statement

No formal ethical review was conducted as positive reference samples were obtained from CCT reactor animals at the slaughterhouse after culling as part of the UK bTB control programme, while negative samples tested were remnants of samples taken under the Veterinary Surgeons Act UK for routine diagnostic purposes and formal permission to test for bTB was sought and obtained from the farmer and APHA. All methods were performed in accordance with relevant national and international guidelines and regulations and complied with ARRIVE guidelines.

### 2.3. Reference samples

Reference samples were taken for routine diagnostic purposes either at a time unrelated to bTB skin testing when anamnestic antibody responses would be minimal or absent (non-boosted), or approximately 5–30 days post tuberculin injection for the CCT when anamnestic antibody responses were likely to be developing or optimal (boosted).

Negative non-boosted milk samples (n = 1149) were obtained from 34 herds comprising a range of dairy breeds (British Friesian, Holstein, Holstein Friesian, Brown Swiss cross) from the low-risk areas of the UK (Cumbria, Lancashire, North and West Yorkshire, West Sussex) which had been free of bTB for at least 8 years, and no herds within 10 km had recorded bTB in the previous 12 months (**Table 1**). Boosted milk samples were available from 353 CCT negative animals from 8 herds. Negative milk samples were tested by ELISA for the presence of antibodies to the following pathogens: Map, BVDV, IBRV, FH, BCV, and BRSV.

**Table 1:** Source and characteristics of individual milk reference samples used in the study.

Origin of samples	Number of samples	Number of herds	Age Mean+/- SD years	Reference standard
**Individual milk** **bTB free** **Non-boosted** **UK**	1149	34	4.9 ± 2.1	CCT negative > 8 years from the low-risk area of the UK. No bTB breakdowns within 10 Km in previous12 months. 1129 *Map* ELISA negative; 20 were *Map* ELISA positive.
**Individual milk** **bTB free** **Boosted** **UK**	353	8	N/A^a^	CCT negative from low-risk area of the UK. No recent history of bTB.
**Individual milk** **CCT positive** **Boosted** **UK**	305	39	5.2+/-2.4 (Partial)^b^	CCT reactors from bTB breakdown herds in high-risk areas of the UK.
**Paired milk and serum** **CCT positive** **Boosted** **UK**	199	22	5.3 ± 2.4	Subset of 305 CCT reactors from bTB breakdown herds in high-risk area of the UK.

Milk and serum samples were obtained from UK herds. The number of samples and herds is shown along with the age of the animals used where known. The criteria used for sample selection is shown. Samples were obtained 5–30 days post a CCT (Boosted) or outside this anamnestic window (Non-boosted).

^a^Not available.

^b^194 animals/305

Positive serum and milk reference samples were obtained from CCT reactor animals at the abattoir (**Table 1**). Animals showing two consecutive inconclusive reactions in the CCT (2 x IR) were included as a source of positive reference samples (APHA Official Veterinarian instructions available from: http://apha.defra.gov.uk/External_OV_Instructions/TB_Instructions/Skin_Test/Skin_Test_Day_Two.html). The serum and individual milk samples were aliquoted and stored at -20°C until tested. The presence of visible lesions (VL) of tuberculosis at post-mortem was recorded by APHA and the results were made available for the study.

Paired anamnestic serum and milk samples were available from 199 CCT positive animals in the UK (Cornwall, Devon, Somerset, Dorset, Wiltshire). The animals were from 22 herds comprising 11 breeds and 11 crossbreeds.

### 2.4. Enferplex Bovine TB antibody test

The antigens used in the multiplex test were Rv2875 synthetic peptide p6 [32]; PPD-B; recombinant Rv2873; recombinant Rv2875; Bovine TB cocktail; recombinant Rv2031c-Rv1886c fusion protein; recombinant Rv3875-Rv3874 fusion protein; recombinant Rv3874-Rv3875 fusion protein; recombinant Rv2626c; recombinant Rv0251c; recombinant Rv2031c [20]. The multiplex test was performed as described previously for serum samples [20, 21] but modified as follows for milk samples. Milk samples (2 ml) were centrifuged at room temperature for 10 min at 20000g to allow the fat component to separate from the milk supernatant. A pipette was carefully inserted down the inside of the tube to remove the fat-free milk fluid. Defatted milk samples were either tested immediately or after refrigeration for up to 24 hours at 2–8°C, or after freezing -20°C.

Individual milk samples were diluted 1:5 in diluent (Buffer B, Enfer Scientific) and paired serum samples were diluted 1:200 in Buffer B. The results were defined using the Enferplex Bovine TB Macro, based on individual antigen thresholds after subtracting the RLU value obtained from a blank spot. The individual antigen thresholds were set using known positive and negative milk samples during test development. Antibody levels for individual antigens were determined by calculating the signal/cut-off (S/CO) ratio for each antigen. Two threshold settings were established during test development: high sensitivity setting (Hse) with a target specificity of 98.0% and a high specificity setting (Hsp) with a target specificity of 99.5%. The threshold for overall assay positivity was set based on a two—antigen rule, whereby the blanked RLU signals from two or more antigens need to be above their individual antigen thresholds for the sample to be registered as “positive” [20].

### 2.5. Analytical specificity assays

The following commercial ELISA kits were used to measure antibodies to other pathogens in negative reference milk samples from bTB-free herds: *Map*—ID Screen® Paratuberculosis Indirect ELISA (ID.vet); *BVDV*–BVDV Total Ab Test (IDEXX); ID Screen® IBR gE Competition (ID.vet); *FH* (SVANOVIR^®^ F. hepatica-Ab (Svanova Diagnostics).); *BCV*–SVANOVIR^®^ BCV-Ab (Svanova Diagnostics); *BRSV*–SVANOVIR^®^ BRSV-Ab (Svanova Diagnostics).

### 2.6. Repeatability and reproducibility trials

To determine the within-run and between-run, variation, three categories of individual milk samples were used: one milk sample that was negative against all 11 antigens; one milk sample giving strong positivity against multiple antigens; one milk sample dilution for each antigen giving weak positivity against multiple antigens. The samples were run in quadruplicate over 20 runs, split between two days and two operators.

For reproducibility studies, an evaluation panel of samples comprising negative, weak positive and strong positive milk samples were blinded and sent to three independent laboratories for reproducibility testing. Seven negative samples, 7 weak positive samples, and 7 strong positive samples (based on the two-antigen rule) were tested in duplicate using two plates from two different kit batches and one technician in each of the three independent laboratories: Laboratory 1. Department of Agriculture, Food & the Marine (DFAM) (ISO17025:2005), Backweston Laboratory Complex, Youngs Cross, Ballymadeer, Celbridge, Co. Kildare, W23 X3PH. Laboratory 2. TINE Norwegian Dairies Mastitis Laboratory (ISO17025 accredited), Post box 2038, 6402 Molde, Norway. Laboratory 3. ALT/Merieux NutriSciences, Biological Testing Laboratory (ISO17025 accredited), Unit 4, Newbridge Industrial Estate, Newbridge, Co, Kildare, Ireland. The results from the three laboratories were sent to Enfer Scientific for un-blinding and statistical analysis.

### 2.7. Statistical analyses

Data were expressed as mean, SD, and 95% confidence interval using Graphpad Prism v9 statistical package. Differences in proportions were assessed using Fisher’s Exact test. The degree of agreement between Enferplex test and positive and negative reference comparators was assessed using Cohen’s Kappa analysis. Marginal homogeneity analysis of results obtained with paired serum and milk was assessed using the McNemar test. Likelihood Ratio analysis was performed on data from CCT positive boosted animals and from CCT positive boosted animals with lesions using Medcalc statistical package. Positive (LR+) and negative (LR-) likelihood values, and diagnostic odds ratio (DOR) with 95% confidence interval (95% CI) were calculated. Spearman rank correlation analysis between paired serum and milk samples from CCT positive animals for the 11 antigens was also carried out.

For the repeatability and reproducibility analyses, a series of linear mixed-effect models were run with operator, day, microtitre plate and sample being entered as random effects. Similar models were run for reproducibility variation due to batch, laboratory, microtitre plate and sample assessed through calculation of intra-class correlation coefficients (ICCs). The results included calculation of the coefficient of variation (CV) defined as the ratio of the SD to the mean expressed as a percentage (%CV). These latter ICC analyses were carried out in R (v3.51, (C) 2018 The R Foundation for Statistical Computing), using the *lme4* (v1.1–18.1), and *sjPlot* (v2.6.0) packages.

## 3. Results

### 3.1 Diagnostic specificity of the Enferplex Bovine TB Antibody test in individual milk samples

Dsp estimates were made using milk samples from bTB-free animals (**Table 2 and S1 Table**). The Dsp of the test was 99.7% at the Hse setting and 99.8% at the Hsp setting. Boosted samples from bTB free herds gave a Dsp of 98.6% and 99.2% at the Hse and Hsp settings of the test respectively. Analytical specificity was assessed using 1149 bTB-free milk samples tested for the presence of antibodies to Map, BVDV, IBRVge, FH, BCV or BRSV. The Enferplex bTB antibody test Dsp remained high when tested in samples positive for antibodies to these pathogens.

**Table 2:** Diagnostic specificity of the Enferplex Bovine TB antibody test using individual milk samples from UK cattle.

Test method under evaluation	Statistical variable	High Sensitivity	High Specificity
**Diagnostic specificity** **CCT and/or OTF status and Bovine TB history** **Non boosted**	Number Dsp 95% CI	1149 99.7% 99.2–99.9	1149 99.8% 99.4–99.9
**Diagnostic specificity** **CCT and/or OTF status and Bovine TB history** **Boosted**	Number Dsp 95% CI	353 98.6% 96.7–99.4	353 99.2% 99.5–99.7
**Map positive** **Non-boosted**	Number Dsp 95% CI	102 99.0% 94.7–99.8	102 99.0% 94.7–99.8
**BVDV positive** **Non-boosted**	Number Dsp 95% CI	611 99.5% 98.6–99.8	611 99.7% 98.8–99.9
**IBRVgE positive** **Non-boosted**	Number Dsp 95% CI	861 99.7% 99.0–99.9	861 99.8% 99.2–99.9
**FH positive** **Non-boosted**	Number Dsp 95% CI	286 99.3% 97.5–99.8	286 99.3% 97.5–99.8
**BCV positive** **Non-boosted**	Number Dsp 95% CI	536 99.8% 99.0–99.8	536 100% -
**BRSV positive** **Non-boosted**	Number Dsp 95% CI	1096 99.7% 99.2–99.9	1096 99.8% 99.3–99.9

Milk samples were obtained from bTB-free UK herds. The samples were tested for antibodies to the following pathogens: Map, BVDV, IBRV, FH, BCV and BRSV. The samples were tested for bTB using the Enferplex Bovine TB antibody test using the Hse and Hsp settings of the test to estimate diagnostic specificity (Dsp) and 95% confidence intervals (95% CI). The criteria used for sample selection is shown. Samples were obtained 5–30 days post a CCT (Boosted) or outside this anamnestic window (Non-boosted).

### 3.2. Relative diagnostic sensitivity of the Enferplex Bovine TB Antibody test in individual milk samples

The relative sensitivity (Rse) and diagnostic sensitivity (Dse) estimates of the Enferplex test using individual milk samples from CCT positive animals were estimated using samples from bTB breakdown herds (**Table 3 and S1 Table**). The results show that the Rse of the Enferplex Bovine TB antibody in boosted samples (n = 305) was 90.8% and 87.2% at the Hse and Hsp settings of the test respectively. The equivalent figures for 18 non-boosted samples were 83.3% and 77.8% respectively. In boosted samples from animals with lesions, the Rse was 96.3% at the Hse setting and 91.3% at the Hsp setting. All 16 samples from *M*. *bovis* culture positive animals were positive (100%) at both settings of the test.

**Table 3:** Relative or diagnostic sensitivity of the Enferplex Bovine TB antibody test using individual milk samples from UK cattle.

Test method under evaluation	Statistical variable	High Sensitivity	High Specificity
**Relative sensitivity** **CCT positive** **Boosted**	Number Rse 95% CI	305 90.8% 87.1–93.6	305 87.2% 83.0–90.6
**Relative sensitivity** **CCT positive** **Non boosted**	Number Rse 95% CI	18 83.3% 60.8–94.2	18 77.8% 54.8–91.0
**Relative sensitivity** **CCT positive,** **bTB lesion positive** **Boosted**	Number Rse 95% CI	80 96.3% 89.6–98.7	80 91.3% 83.0–95.7
**Diagnostic sensitivity. *M*. *bovis* culture positive** **Boosted**	Number Dse 95% CI	16 100% -	16 100% -
**Relative sensitivity** **All samples**	Number Rse 95% CI	325 89.9% 86.1–92.7	325 85.9% 81.6–89.2

Milk samples were obtained from UK CCT positive animals from the UK. The samples were tested using the Enferplex Bovine TB antibody test using the Hse and Hsp settings of the test to estimate relative sensitivity (Rse) and 95% confidence intervals (95% CI). The criteria used for sample selection is shown. Samples were obtained 5–30 days post a CCT (Boosted) or outside this anamnestic window (Non-boosted).

Agreement analysis of boosted CCT positive samples and bTB-free samples using the Hse setting gave a Kappa value of 0.934 (95% CI: 0.911–0.957) indicating almost perfect agreement. Similarly, a Kappa value of 0.957 (95% CI: 0.923–0.991) was found using boosted CCT positive lesioned animals and bTB-free samples at the Hse setting, indicating almost perfect agreement.

The results of marginal homogeneity analysis of 199 paired samples (**Table 4**) show that the differences in proportions between serum and milk were not statistically significant at either the high sensitivity setting (χ^2^_df = 1_ = 1.500, P = 0.227) or the high specificity setting of the test (χ^2^_1_ = 0.444, P = 0.505).

**Table 4:** Comparison of proportions obtained between boosted paired serum and milk samples from CCT positive cattle using the Enferplex Bovine TB antibody test.

Sample	CCT positive cattle	High sensitivity setting			High specificity setting		
		Number of animals			Number of animals		
		Serum		Total	Serum		Total
	Result	Positive	Negative		Positive	Negative	
**Milk**	**Positive**	179	5	184	171	3	174
	**Negative**	1	14	15	6	19	25
**Total**		180	19	199	177	22	199

Results obtained from the Enferplex Bovine TB antibody test in paired serum and milk samples from CCT positive animals were analysed using McNemar’s marginal homogeneity test. Results obtained using the Hse and Hsp settings of the Enferplex Bovine TB antibody test are shown.

Likelihood ratio analysis gave values LR+ and LR^-^ values of 347.8 and 0.09 respectively for boosted samples from CCT positive animals (**Table 5**). The diagnostic odds ratio (DOR) was 3779. The LR+ and LR- were 367.7 and 0.048 respectively for boosted samples from SICCT positive animals with lesions. The DOR was 9168. Test outputs with an LR^+^ > 10 or LR^-^ < 0.1 are considered good diagnostic evidence of the infection being either present or absent respectively [32]. The results show that the LR^+^ was > 10 and the LR^-^ was 0.1 or less, indicating that the Enferplex Bovine TB antibody test results provide good diagnostic evidence of the infection being either present or absent respectively. The DOR provides an overall assessment measure of the test and indicates a good test performance showing values well above 100, a cut-off obtained by dividing an LR+ value = 10 with an LR- value of 0.1.

**Table 5:** Likelihood ratio (LR) for positive (LR+) and negative (LR-) individual milk samples.

Test method under evaluation	Rse^a^ 95% CI	Dsp^b^ 95% CI	LR+^c^ 95% CI	LR-^d^ 95% CI	DOR^e^
**CCT positive** **Boosted milk**	90.8% 87.1–93.6	99.7% 99.2–99.9	347.8 112.3–1077.5	0.09 0.07–0.13	3779 1141–12521
**CCT positive,** **bTB lesion positive** **Boosted milk**	96.0% 88.9–98.6	99.7% 99.2–99.9	367.7 118.7–1139.4	0.05 0.02–0.13	9168 1818–46232

Results obtained using the Enferplex Bovine TB antibody test at the Hse setting were assessed using likelihood ratio analysis. Rse and Dsp estimates using boosted samples and the Hse setting of the test shown in Tables 2 and 3 were used in the LR analyses. 95% confidence intervals (95% CI) are shown.

^a^Rse—Relative sensitivity; ^b^Dsp—Diagnostic specificity; ^c^LR+—Positive likelihood ratio; ^d^LR-—Negative likelihood ratio; ^e^DOR—Diagnostic odds ratio.

### 3.3. Correlation between paired milk and serum samples

**Fig 2:**
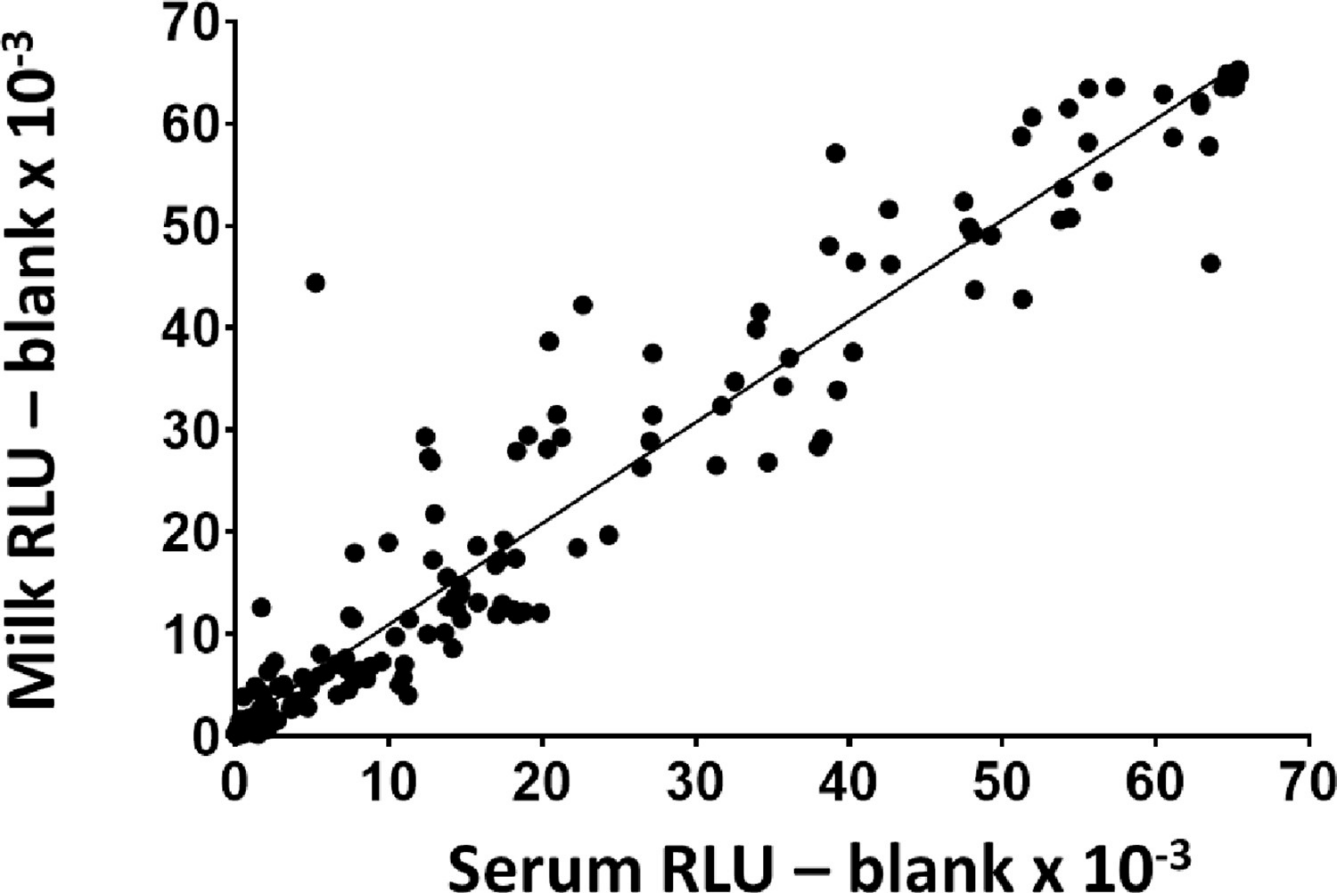
Correlation between paired serum and milk in SICCT positive animals for test antigen PPDb using the Enferplex Bovine TB antibody test. The relative light unit (RLU) obtained with the blank spot was subtracted from the RLU value obtained from antigen spots to obtain a blanked RLU value for each serum and milk sample. Results obtained from serum are shown on the X axis and from the milk samples are shown on the Y axis. Spearman’s rank correlation coefficient r was 0.909, P < 0.0001.

A correlation analysis was performed between the 199 paired serum and milk samples from CCT positive animals (**S1 Fig**) and for illustration purposes the results for PPDb antigen are shown in **Fig 2** with RLU values x 10^−3^ for serum on X axis and for milk on Y axis. Statistically significant positive correlation coefficients obtained in paired serum and milk samples for each antigen which ranged between 0.779–0.955 across the 11 antigens (**Table 6**). The results show that there was a strong positive correlation between the serum and milk results, with only a few outliers found for individual antigens.

**Table 6:** Spearman’s rank correlation coefficient (ρ), 95% confidence intervals, and associated p-values between serum and milk obtained in the Enferplex Bovine TB antibody test using boosted samples.

Antigen^a^	ρ^b^	95% CI^c^	P value
**Rv2875 peptide**	0.932	0.912–0.948	P < 0.0001
**PPDb**	0.909	0.882–0.931	P < 0.0001
**Rv2873**	0.908	0.880–0.929	P < 0.0001
**Rv2875**	0.936	0.917–0.951	P < 0.0001
**Bovine cocktail**	0.955	0.940–0.965	P < 0.0001
**Rv2031c-Rv1886c fusion**	0.856	0.814–0.889	P < 0.0001
**Rv3875-Rv3874 fusion**	0.787	0.727–0.834	P < 0.0001
**Rv3874-Rv3875 fusion**	0.756	0.690–0.810	P < 0.0001
**Rv2626c**	0.782	0.722–0.831	P < 0.0001
**Rv0251c**	0.834	0.786–0.872	P < 0.0001
**Rv2031c**	0.779	0.718–0.828	P < 0.0001

^a^PPDb—bovine purified protein derivative; Bovine cocktail (Lionex); ^b^ρ **−** Spearman’s rank correlation coefficient; 95% confidence interval.

**Fig 3:**
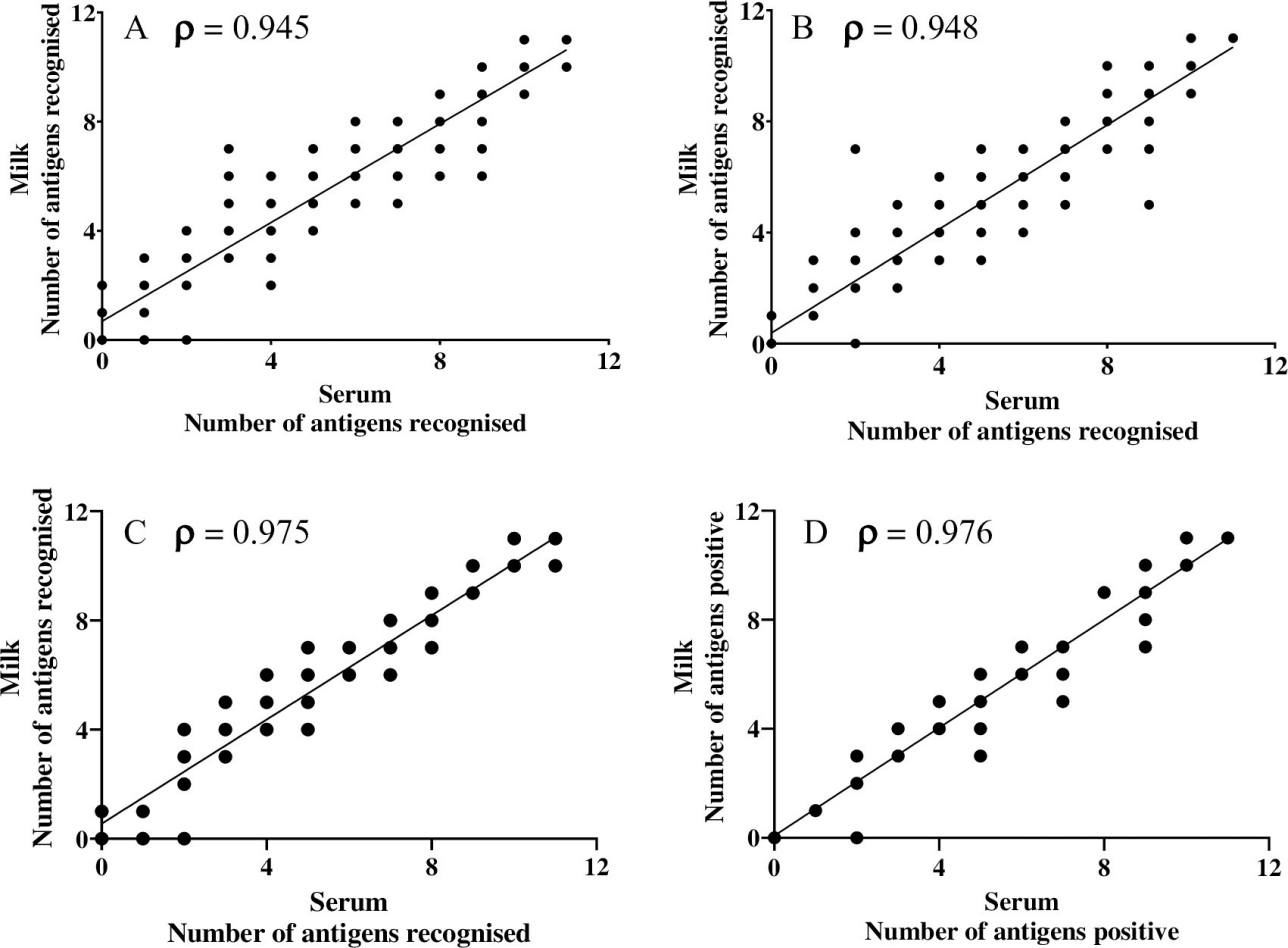
Correlation analysis of the number of test antigens recognised by antibody in paired serum and milk from 199 CCT test positive animals. A—CCT test positive, Hse setting; B—CCT test positive, Hsp setting; C—CCT test positive with VL, Hse setting; D—CCT test positive with VL, Hsp setting. p—Spearman’s Rank Correlation Coefficient. In all 4 comparisons, P values were < 0.0001.

The number of antigens recognised by antibody in the Enferplex Bovine TB antibody test was scored for each paired serum and milk sample and compared (**Fig 3**). Analysis of samples from CCT positive animals gave a correlation coefficient of 0.945 (95% CI: 0.927–0.960) and 0.948 (95% CI: 0.932–0.962) using the Hse and Hsp setting respectively. When serum and milk from a subset of animals that had VL at post-mortem were compared using the Hse and Hsp settings of the test, correlation coefficients of 0.975 (95% CI: 0.959–0.985) and 0.976 (95% CI: 0.960–0.986) were obtained respectively. P values were P < 0.0001 for all four comparisons. The results show that milk maintains the same rank order of number of antigens recognised by antibody as serum.

### 3.4. Repeatability of the Enferplex Bovine TB antibody test using individual milk samples

The variation in S/CO ratios within-run and between-run were determined. The within-run %CVs for the 11 antigens ranged between 2.0 and 3.9% for the strong positive sample, and between 3.8–9.8 for the weak positive sample. Similarly, the between-run CVs ranged between 1.4 and 3.3% for the strong positive sample, and between 3.2–11.1% for the weak positive sample. The %CV ranges observed between two operators for the strong and weak positive samples were 1.4–3.5% and 3.4–10.6% respectively. The %CVs for the negative sample were not determined due to the S/CO ratios being around or below zero, rendering %CVs meaningless. Analysis of the variation observed across 20 runs of the test showed 654/660 (99.1%) data points were less than 2 SDs above or below the mean values across the 11 antigen spots. The ICC analysis showed that < 1% of the variation observed was due to the test plate, day, or operator.

### 3.5. Reproducibility of the Enferplex Bovine TB antibody test using individual milk samples

To assess the reproducibility of the Enferplex bTB antibody test, 7 negative, 7 weak positive and 7 strong positive milk samples were tested in duplicate using two kit batches in three independent laboratories.

#### 3.5.1 Analytical reproducibility

The mean S/CO ratio values obtained for the negative samples were all close to zero (**S2 Fig**). Most S/CO ratio responses (208/231) obtained with weak positive samples had CVs less than 10%. There were 23 exceptions where %CVs were > 10%. Of these, 17/23 samples were associated with responses that were below the threshold for the individual antigens and would be deemed to be negative responses for those antigens. The remaining 6/23 samples had %CVs ranging from 10.4%– 12.6%.

Most S/CO ratio responses (224/231) obtained with strong positive samples had %CVs less than 10%. There were 8 exceptions where %CVs were > 10%. Of these, 5/8 samples were associated with responses that were below the threshold for the individual antigens and would be deemed to be negative responses for those antigens. The remaining 3/8 samples had %CVs ranging from 12.1%– 15.9%. The ICC analyses showed that >99% of the variation observed was due to the sample and 1% due to the kit batch or laboratory.

#### 3.5.2. Diagnostic reproducibility

Diagnostic reproducibility was assessed in three independent laboratories using 7 negative, 7 weak positive and 7 strong positive milk samples tested in duplicate. The results showed complete concordance between the 3 laboratories, with all categories of sample.

## 4. Discussion

We have shown previously that multiplexing *M*. *bovis* antigens allowed the development of a serological assay for bTB that is not only highly sensitive and specific but shows good repeatability and reproducibility [20]. The test is based on a microarray of 11 bTB antigen spots and a chemiluminescent endpoint for each spot. The combination of multiple antigens and chemiluminescence provides an efficient means to attaining high sensitivity. To achieve high specificity, a two-antigen rule was used to classify results whereby two or more antigen spots must give signals above their individual thresholds before the samples was deemed to be ‘positive’ [21].

The use of serum as medium allows high throughput analysis for surveillance and eradication purposes. However, collection of serum samples is not always feasible due to cost, lack of resources and other logistic constraints, and many veterinary serological diagnostic assays have been adapted for use in milk samples which are easier and cheaper to collect and are often taken routinely for monitoring quality and disease control purposes.

While individual milk assays are available for many pathogens, very few studies have been performed to assess the potential use of milk in bTB diagnosis [33, 34]. In this report, we have adapted the Enferplex Bovine TB antibody test for use in bovine milk and assessed the diagnostic accuracy of the test using individual samples. We have used the CCT as the main reference standard due to its high specificity [5] and its use as the main disclosure test for bTB in UK, IE and other countries to determine infected herds. Anamnestic milk samples were used in the study since it is well established in the literature that serum antibody levels are elevated following boosting with tuberculin [10, 13, 14, 35, 36], and it was likely that a similar boosting effect would be manifest using milk samples.

The Dsp of the Enferplex bTB milk test was observed to be 99.7% using the Hse setting of the test in UK dairy herds using non-anamnestic samples, and 98.5% using anamnestic samples. When samples from bTB-free cattle with evidence of infection with non-tuberculous pathogens such as Map, IBRV, BVDV, FH, BCV and BRSV were analysed, the Dsp remained high, showing that these infections did not influence the accuracy of the Enferplex bTB antibody test in bTB-free animals. These results are thus consistent with those found using serum at the Hse setting [20].

The Rse of the test was found to be 90.8% in boosted samples using the Hse setting and 96.3% in animals showing VL at post-mortem. A positive correlation between serum antibody and lesions in tuberculin positive animals is well documented in the literature [20, 35, 37, 38] and this result using milk is consistent with those studies. These results compare well with a Rse (87.8%) and Dsp (97.7%) found in milk from Korean cattle using by ELISA [33]. In contrast, the Enferplex test results show a higher performance compared to the Rse (50%) and Dsp (97.5%) reported using a commercially available bTB antibody test in bovine milk from *M*. *bovis* culture positive animals [34].

When the Enferplex test results obtained using paired serum and milk were compared, good positive correlations were observed for all 11 antigen spots. The Spearman rank correlation coefficients ranged from 0.78–0.96 across all antigens. These results are comparable to a correlation coefficient of 0.83 for a bTB antibody ELISA between milk and serum, and antibody tests in cattle for *BVDV* or *NC* infections where correlation coefficients of 0.925 and 0.702 were reported respectively [30, 33, 39].

The high positive correlation values found for individual antigens in the Enferplex milk test was reflected in the high correlation coefficients observed when the number of antigens recognised by antibody was compared in serum and milk samples. Consistent with this, agreement analysis gave high Kappa values at the two sensitivity settings of the test and application of the McNemar discriminant test indicated no statistically significant differences between serum and milk results. The agreement test result obtained overall using the Enferplex Bovine TB antibody test (Kappa = 0.934) is consistent with those in the literature for *BVDV* (Kappa = 0.865) but is considerably higher than for those observed in *Map* infection (Kappa = 0.500) [25, 31].

The reasons why the correlation is so poor in Johne’s disease compared to *BVDV* and Enferplex Bovine TB antibody tests are unknown. Poor *Map* test sensitivity may play a role as IgG concentrations are much lower in milk than serum [40]. It is known that normal bovine serum contains both IgG1 and IgG2 subclasses at similar concentrations, while in milk the IgG1 subclass dominates [40–43]. IgG1 fixes complement, but IgG2 is associated with opsonisation for removal and destruction of bacteria by macrophages [43]. IFNγ is also known to upregulate IgG2 responses [43], suggesting that TH1 responses in animals with Johnes may influence the IgG1/IgG2 balance in blood leading to discrepancies with milk responses. However, IgG1 responses to PPDa have been shown to increase in the clinical stage of Johnes compared to asymptomatic stage, while IgG1 and IgG2 responses to other antigens such as Hsp70, Hsp65 and LAM were decreased in the clinical versus asymptomatic stages [44]. This suggests that discrepancies between IgG1 and IgG2 antibody responses in Johne’s disease may be dependent on antibody responses to particular Map antigens.

In bTB, IgG1 anti-MPB70 antibody levels in animals with bTB are higher in animals that show VL at post-mortem and are *M*. *bovis* culture positive [45]. In the latter study, no individual MPB70 epitopes were exclusively recognised by IgG1 or IgG2 antibodies. However, IgG1 and IgG2 serum antibody responses in *M*. *bovis* experimentally infected animals have been shown to be similar in some animals but to have higher IgG1 antibody levels in others [37]. It is possible that fluctuations in IFNγ responses may have resulted in the different IgG subclass responses observed in the latter study. Despite these variations in IgG isotype responses, we found that the Rse of the Enferplex antibody test in boosted milk samples from CCT positive animals was comparable to that previously observed in serum using Hse setting [20]. It is possible that the dominance of IgG1 responses masked contributions from IgG2 antibodies such that little difference between serum and milk responses is observed. Alternatively, boosting with tuberculin may increase some antibody responses over others which could mask isotype differences. Analysis of IgG subclass levels in paired serum and milk samples would be required to determine if any of these possibilities are correct or not.

We performed likelihood ratio analysis to assess the likelihood of the Enferplex milk test results being true versus false across both infected and non-infected individuals. Positive LR values that are above 10 and negative LR values that are below 0.1 are deemed to provide good diagnostic evidence for infection being present or absent respectively [32]. The likelihood ratio analysis showed that the positive and negative LR values for the Enferplex Bovine TB antibody test were well above and below these limits respectively, indicating the fitness-for-purpose of the Enferplex milk test for ruling in and ruling out bTB in cattle.

Collectively, these results show excellent comparability between milk and serum results and indicate that individual milk samples could substitute for serum while retaining high sensitivity and specificity for detecting bTB. It should be noted, however, that various non-disease factors have been shown to affect diagnostic test results when milk is used as the medium. These include milk yield, protein concentrations, presence of inhibitors, parity, and stage of lactation [46–50], though a recent analysis of results from *Map* infected cattle found that these variables explained only 5.1% of the variation observed in antibody levels [51]. Another study found that there were no production losses associated with any of these variables [52]. We have not determined whether such factors affect the Enferplex bTB antibody test in this report, but it will be important to assess these potential effects in further studies.

## 5. Conclusions

This report shows that the Rse and Dsp of the Enferplex bovine TB antibody test are both high using individual milk as the sample specimen and demonstrates good agreement between milk antibody results and CCT status. Strong positive correlations were observed between serum and milk antibody responses to each of the 11 antigens in the test, and with the number of antigens recognised by antibody. The LR+ and LR- values obtained using milk provide good diagnostic evidence of the infection being either present or absent respectively. The test also exhibits good repeatability and reproducibility. The results show that individual milk samples can be used in the Enferplex Bovine TB antibody test instead of serum for the diagnosis of bTB.

## Supporting information

S1 Table Raw data used to estimate relative sensitivity and diagnostic specificity. The status of the CCT and presence of VL are indicated.
(PDF)

S1 Fig Correlation between serum and milk results for each antigen used in the test. The relative light unit (RLU) obtained with the blank spot was subtracted from the RLU value obtained from antigen spots to obtain a blanked RLU value for each serum and milk sample. Results obtained from serum are shown on the Y axis and from the milk samples are shown on the X axis. Spearman’s rank correlation coefficient ρ obtained with paired serum and milk samples for each antigen ranged between 0.779–0.955 across the 11 antigens (P <0.0001 for all antigens).
(PDF)

S2 Fig Reproducibility plots of test results obtained by three independent laboratories. An evaluation panel of samples comprising negative, weak positive and strong positive milk samples were blinded and sent to three independent laboratories for reproducibility testing. Seven negative samples, 7 weak positive samples, and 7 strong positive samples (based on the two-antigen rule) were tested in duplicate using two plates from two different kit batches and one technician in each of the three independent laboratories. The results obtained for raw and signal/cut-off data are shown in the plots.
(PDF)
