# Supplementary material for: Diagnostic accuracy of the Enferplex Bovine TB antibody test using individual milk samples from cattle
Source: PLoS One. 2024 Apr 30;19(4):e0301609. doi: 10.1371/journal.pone.0301609 (PMC11060599; doi:10.1371/journal.pone.0301609)
Supplement: S1 Table — The status of the CCT and presence of VL are indicated. (PDF) [file pone.0301609.s001.pdf]

S1 Table. Data used to estimate relative sensitivity and diagnostic specificity.

Data obtained with positive and negative reference milk samples showing the number positive and the number of antigens recognised by antibody. The test results obtained using the high sensitivity (Hse) and high specificity (Hsp) settings of the test are included along with the skin test and postmortem results.

Reference sample	Animal ID	Number of antigens recognised	Hse Result	Number of antigens recognised	Hsp Result	Skin Test Result	Anamnestic Boost	PM Results	Number of Lesions
Positive	**MSBTB MP003**	8	Positive	7	Positive	2xIR	Yes	NVL	0
Positive	**MSBTB MP004**	10	Positive	8	Positive	2xIR	Yes	NVL	0
Positive	**MSBTB MP005**	9	Positive	9	Positive	R	Yes	VL	1
Positive	**MSBTB MP006**	5	Positive	4	Positive	R	Yes	NVL	0
Positive	**MSBTB MP007**	7	Positive	7	Positive	R	Yes	VL	1
Positive	**MSBTB MP008**	9	Positive	9	Positive	R	Yes	NVL	0
Positive	**MSBTB MP009**	8	Positive	7	Positive	R	Yes	NVL	0
Positive	**MSBTB MP010**	0	Negative	0	Negative	R	Yes	VL	2
Positive	**MSBTB MP011**	7	Positive	6	Positive	R	Yes	VL	1
Positive	**MSBTB MP012**	5	Positive	5	Positive	R	Yes	NVL	0
Positive	**MSBTB MP013**	3	Positive	3	Positive	R	Yes	NVL	0
Positive	**MSBTB MP014**	10	Positive	10	Positive	R	Yes	VL	5
Positive	**MSBTB MP015**	10	Positive	9	Positive	R	Yes	VL	2
Positive	**MSBTB MP016**	5	Positive	5	Positive	R	Yes	NVL	0
Positive	**MSBTB MP017**	2	Positive	1	Negative	R	Yes	NVL	0
Positive	**MSBTB MP018**	9	Positive	9	Positive	R	Yes	VL	1
Positive	**MSBTB MP019**	9	Positive	9	Positive	R	Yes	VL	1
Positive	**MSBTB MP020**	5	Positive	5	Positive	R	Yes	VL	1
Positive	**MSBTB MP022**	5	Positive	5	Positive	R	Yes	NVL	0

Positive	**MSBTB MP023**	10	Positive	7	Positive	R	Yes	VL	1
Positive	**MSBTB MP026**	2	Positive	2	Positive	R	Yes	NVL	0
Positive	**MSBTB MP027**	10	Positive	10	Positive	R	Yes	NVL	0
Positive	**MSBTB MP028**	10	Positive	9	Positive	R	Yes	NVL	0
Positive	**MSBTB MP029**	7	Positive	5	Positive	R	Yes	VL	1
Positive	**MSBTB MP030**	8	Positive	5	Positive	R	Yes	NVL	0
Positive	**MSBTB MP031**	5	Positive	5	Positive	R	Yes	NVL	0
Positive	**MSBTB MP032**	5	Positive	5	Positive	R	Yes	VL	1
Positive	**MSBTB MP033**	5	Positive	5	Positive	R	Yes	NVL	0
Positive	**MSBTB MP034**	5	Positive	5	Positive	R	Yes	VL	1
Positive	**MSBTB MP035**	4	Positive	2	Positive	R	Yes	VL	1
Positive	**MSBTB MP036**	11	Positive	11	Positive	R	Yes	NVL	0
Positive	**MSBTB MP037**	10	Positive	10	Positive	R	Yes	NVL	0
Positive	**MSBTB MP038**	1	Negative	1	Negative	R	Yes	NVL	0
Positive	**MSBTB MP039**	5	Positive	5	Positive	R	Yes	NVL	0
Positive	**MSBTB MP040**	11	Positive	11	Positive	R	Yes	VL	1
Positive	**MSBTB MP042**	6	Positive	6	Positive	R	Yes	NVL	0
Positive	**MSBTB MP058**	5	Positive	5	Positive	R	Yes	NVL	0
Positive	**MSBTB MP059**	10	Positive	10	Positive	R	Yes	NVL	0
Positive	**MSBTB MP060**	10	Positive	10	Positive	R	Yes	NVL	0
Positive	**MSBTB MP061**	10	Positive	10	Positive	R	Yes	VL	1
Positive	**MSBTB MP062**	5	Positive	5	Positive	R	Yes	VL	1
Positive	**MSBTB MP063**	1	Negative	1	Negative	R	Yes	NVL	0
Positive	**MSBTB MP078**	4	Positive	2	Positive	R	Yes	NVL	0
Positive	**MSBTB MP080**	10	Positive	9	Positive	R	Yes	VL	1
Positive	**MSBTB MP088**	10	Positive	10	Positive	R	Yes	VL	1
Positive	**MSBTB MP089**	7	Positive	7	Positive	R	Yes	VL	2
Positive	**MSBTB MP090**	1	Negative	0	Negative	2xIR	Yes	NVL	0
Positive	**MSBTB MP092**	6	Positive	6	Positive	R	Yes	NVL	0
Positive	**MSBTB MP096**	4	Positive	3	Positive	R	Yes	NVL	0
Positive	**MSBTB MP097**	10	Positive	10	Positive	R	Yes	NVL	0

Positive	**MSBTB MP098**	6	Positive	6	Positive	R	Yes	NVL	0
Positive	**MSBTB MP100**	6	Positive	6	Positive	R	Yes	NVL	0
Positive	**MSBTB MP101**	7	Positive	5	Positive	R	Yes	NVL	0
Positive	**MSBTB MP102**	8	Positive	8	Positive	R	Yes	NVL	0
Positive	**MSBTB MP103**	10	Positive	8	Positive	R	Yes	VL	1
Positive	**MSBTB MP104**	6	Positive	5	Positive	R	Yes	NVL	0
Positive	**MSBTB MP105**	3	Positive	2	Positive	R	Yes	NVL	0
Positive	**MSBTB MP111**	1	Negative	1	Negative	R	Yes	NVL	0
Positive	**MSBTB MP112**	9	Positive	9	Positive	R	Yes	VL	1
Positive	**MSBTB MP113**	1	Negative	0	Negative	2xIR	Yes	NVL	0
Positive	**MSBTB MP114**	5	Positive	4	Positive	R	Yes	NVL	0
Positive	**MSBTB MP115**	5	Positive	3	Positive	R	Yes	NVL	0
Positive	**MP119 Repeat**	4	Positive	4	Positive	2xIR	Yes	NVL	0
Positive	**MSBTB MP122**	9	Positive	7	Positive	R	Yes	NVL	0
Positive	**MSBTB MP126**	0	Negative	0	Negative	R	Yes	NVL	0
Positive	**MSBTB MP129**	4	Positive	2	Positive	R	Yes	NVL	0
Positive	**MSBTB MP132**	11	Positive	11	Positive	R	Yes	VL	2
Positive	**MSBTB MP133**	8	Positive	7	Positive	R	Yes	VL	1
Positive	**MSBTB MP141**	10	Positive	9	Positive	R	Yes	NVL	0
Positive	**MSBTB MP142**	10	Positive	9	Positive	R	Yes	NVL	0
Positive	**MSBTB MP143**	3	Positive	2	Positive	R	Yes	NVL	0
Positive	**MSBTB MP144**	2	Positive	1	Negative	R	Yes	NVL	0
Positive	**MSBTB MP145**	5	Positive	3	Positive	R	Yes	NVL	0
Positive	**MSBTB MP150**	6	Positive	6	Positive	R	Yes	NVL	0
Positive	**MSBTB MP151**	7	Positive	6	Positive	R	Yes	NVL	0
Positive	**MSBTB MP154**	6	Positive	6	Positive	R	Yes	NVL	0
Positive	**MSBTB MP164**	8	Positive	7	Positive	R	Yes	NVL	0
Positive	**MSBTB MP166**	1	Negative	0	Negative	R	Yes	NVL	0
Positive	**MSBTB MP168**	6	Positive	6	Positive	R	Yes	NVL	0
Positive	**MSBTB MP172**	4	Positive	2	Positive	R	Yes	VL	1
Positive	**MSBTB MP175**	7	Positive	6	Positive	R	Yes	NVL	0

Positive	**MSBTB MP176**	10	Positive	9	Positive	R	Yes	NVL	0
Positive	**MSBTB MP190**	2	Positive	1	Negative	R	Yes	NVL	0
Positive	**MSBTB MP192**	4	Positive	4	Positive	R	Yes	NVL	0
Positive	**MSBTB MP194**	2	Positive	2	Positive	R	Yes	VL	1
Positive	**MSBTB MP197**	3	Positive	3	Positive	R	Yes	NVL	0
Positive	**MSBTB MP201**	2	Positive	2	Positive	R	Yes	NVL	0
Positive	**MSBTB MP202**	10	Positive	10	Positive	R	Yes	NVL	0
Positive	**MSBTB MP203**	6	Positive	5	Positive	R	Yes	VL	1
Positive	**MP212Repeat**	6	Positive	6	Positive	R	Yes	NVL	0
Positive	**MSBTB MP213**	3	Positive	2	Positive	R	Yes	VL	2
Positive	**MSBTB MP214**	6	Positive	6	Positive	R	Yes	NVL	0
Positive	**MSBTB MP216**	5	Positive	5	Positive	R	Yes	NVL	0
Positive	**MSBTB MP217**	6	Positive	6	Positive	R	Yes	NVL	0
Positive	**MSBTB MP218**	8	Positive	6	Positive	R	Yes	NVL	0
Positive	**MSBTB MP219**	8	Positive	7	Positive	R	Yes	NVL	0
Positive	**MSBTB MP220 Repeat**	6	Positive	6	Positive	R	Yes	NVL	0
Positive	**MSBTB MP221**	3	Positive	2	Positive	R	Yes	NVL	0
Positive	**MSBTB MP222**	0	Negative	0	Negative	R	Yes	NVL	0
Positive	**MSBTB MP223 Repeat**	0	Negative	0	Negative	R	Yes	VL	N/A
Positive	**MSBTB MP224**	8	Positive	8	Positive	R	Yes	VL	N/A
Positive	**MSBTB MP225**	3	Positive	3	Positive	R	Yes	NVL	0
Positive	**MSBTB MP226**	6	Positive	6	Positive	R	Yes	NVL	0
Positive	**MSBTB MP228**	7	Positive	7	Positive	R	Yes	VL	N/A
Positive	**MSBTB MP229**	5	Positive	4	Positive	R	Yes	VL	N/A
Positive	**MSBTB MP230**	5	Positive	5	Positive	R	Yes	NVL	0
Positive	**MSBTB MP231**	10	Positive	10	Positive	R	Yes	VL	N/A
Positive	**MSBTB MP232**	9	Positive	9	Positive	R	Yes	VL	N/A
Positive	**MSBTB MP233 Repeat**	10	Positive	10	Positive	R	Yes	VL	N/A
Positive	**MSBTB MP235**	3	Positive	3	Positive	R	Yes	NVL	0
Positive	**MSBTB MP236 Repeat**	5	Positive	5	Positive	R	Yes	NVL	0
Positive	**MSBTB MP237**	9	Positive	8	Positive	R	Yes	NVL	0

Positive	**MSBTB MP245 Repeat**	1	Negative	1	Negative	R	Yes	NVL	0
Positive	**MSBTB MP248 Repeat**	5	Positive	4	Positive	R	Yes	NVL	0
Positive	**MSBTB MP249**	6	Positive	6	Positive	R	Yes	VL	N/A
Positive	**MSBTB MP250 Repeat**	5	Positive	5	Positive	R	Yes	NVL	0
Positive	**MSBTB MP252 Repeat**	7	Positive	7	Positive	R	Yes	NVL	0
Positive	**MSBTB MP253**	3	Positive	2	Positive	R	Yes	NVL	0
Positive	**MSBTB MP254**	6	Positive	6	Positive	R	Yes	NVL	0
Positive	**MSBTB MP255**	8	Positive	7	Positive	R	Yes	NVL	0
Positive	**MSBTB MP256**	5	Positive	5	Positive	R	Yes	NVL	0
Positive	**MSBTB MP257**	8	Positive	7	Positive	R	Yes	NVL	0
Positive	**MSBTB MP273**	8	Positive	8	Positive	R	Yes	NVL	0
Positive	**MSBTB SP276 Repeat**	4	Positive	4	Positive	R	Yes	NVL	0
Positive	**MSBTB MP277**	3	Positive	2	Positive	R	Yes	NVL	0
Positive	**MSBTB MP278**	11	Positive	11	Positive	R	Yes	NVL	0
Positive	**MSBTB MP296**	9	Positive	8	Positive	R	Yes	VL	N/A
Positive	**MSBTB MP298 Repeat**	3	Positive	3	Positive	R	Yes	VL	N/A
Positive	**MSBTB MP299 Repeat**	5	Positive	5	Positive	R	Yes	NVL	0
Positive	**MSBTB MP300 Repeat**	6	Positive	4	Positive	R	Yes	NVL	0
Positive	**MSBTB MP302 Repeat**	3	Positive	1	Negative	R	Yes	VL	1
Positive	**MSBTB MP303 Repeat**	6	Positive	5	Positive	R	Yes	VL	1
Positive	**MSBTB MP305 Repeat**	5	Positive	4	Positive	R	Yes	NVL	0
Positive	**MSBTB MP310 Repeat**	10	Positive	10	Positive	R	Yes	VL	1
Positive	**MSBTB MP311**	2	Positive	1	Negative	R	Yes	VL	1
Positive	**MSBTB MP312 Repeat**	7	Positive	7	Positive	R	Yes	VL	1
Positive	**MSBTB MP313 Repeat**	8	Positive	7	Positive	R	Yes	NVL	0
Positive	**MSBTB MP314**	5	Positive	4	Positive	R	Yes	VL	1
Positive	**MSBTB MP315**	4	Positive	4	Positive	R	Yes	NVL	0
Positive	**MSBTB MP316 Repeat**	7	Positive	7	Positive	R	Yes	VL	1
Positive	**MSBTB MP317 Repeat**	8	Positive	7	Positive	R	Yes	VL	2
Positive	**MSBTB MP318**	6	Positive	6	Positive	R	Yes	VL	2
Positive	**MSBTB MP319 Repeat**	5	Positive	3	Positive	R	Yes	NVL	0

Positive	**MSBTB MP320**	4	Positive	4	Positive	R	Yes	VL	3
Positive	**MSBTB MP321**	2	Positive	1	Negative	R	Yes	VL	1
Positive	**MSBTB MP322 Repeat**	7	Positive	7	Positive	R	Yes	VL	1
Positive	**MSBTB MP323 Repeat**	3	Positive	3	Positive	R	Yes	VL	1
Positive	**MSBTB MP324**	4	Positive	3	Positive	R	Yes	NVL	0
Positive	**MSBTB MP325**	2	Positive	0	Negative	R	Yes	VL	1
Positive	**MSBTB MP326 Repeat**	6	Positive	6	Positive	R	Yes	VL	1
Positive	**MSBTB MP327 Repeat**	4	Positive	4	Positive	R	Yes	VL	1
Positive	**MSBTB MP328**	5	Positive	4	Positive	R	Yes	NVL	0
Positive	**MSBTB MP329**	6	Positive	6	Positive	R	Yes	VL	1
Positive	**MSBTB MP338 Repeat**	6	Positive	6	Positive	R	Yes	NVL	0
Positive	**MSBTB MP339 Repeat**	7	Positive	7	Positive	R	Yes	NVL	0
Positive	**MSBTB MP340 Repeat**	4	Positive	4	Positive	R	Yes	NVL	0
Positive	**MSBTB MP358 Repeat**	10	Positive	9	Positive	R	Yes	VL	1
Positive	**MSBTB MP362 Repeat**	6	Positive	6	Positive	R	Yes	VL	1
Positive	**MSBTB MP379 Repeat**	5	Positive	3	Positive	R	Yes	NVL	0
Positive	**MSBTB MP383 Repeat**	9	Positive	9	Positive	R	Yes	VL	1
Positive	**MSBTB MP387 Repeat**	9	Positive	9	Positive	R	Yes	NVL	0
Positive	**MSBTB MP390 Repeat**	7	Positive	6	Positive	R	Yes	NVL	0
Positive	**MSBTB MP395 Repeat**	4	Positive	4	Positive	R	Yes	NVL	0
Positive	**MSBTB MP399 Repeat**	2	Positive	1	Negative	R	Yes	NVL	0
Positive	**MSBTB MP401 Repeat**	8	Positive	7	Positive	R	Yes	NVL	0
Positive	**MSBTB SP402 Repeat 10.9.21**	5	Positive	5	Positive	R	Yes	NVL	0
Positive	**MSBTB MP405 Repeat**	0	Negative	0	Negative	R	Yes	NVL	0
Positive	**MSBTB MP406 Repeat**	4	Positive	4	Positive	R	Yes	NVL	0
Positive	**MSBTB MP410**	8	Positive	8	Positive	R	Yes	NVL	0
Positive	**MSBTB MP423 Repeat**	3	Positive	3	Positive	R	Yes	VL	1
Positive	**MSBTB MP426 Repeat**	5	Positive	4	Positive	R	Yes	NVL	0
Positive	**MSBTB MP427**	9	Positive	9	Positive	R	Yes	NVL	0
Positive	**MSBTB MP428**	2	Positive	2	Positive	R	Yes	NVL	0

Positive	**MSBTB MP429**	4	Positive	4	Positive	R	Yes	NVL	0
Positive	**MSBTB MP430**	4	Positive	4	Positive	R	Yes	NVL	0
Positive	**MSBTB MP431**	3	Positive	1	Negative	R	Yes	NVL	0
Positive	**MSBTB MP432**	4	Positive	3	Positive	R	Yes	NVL	0
Positive	**MSBTB MP433**	5	Positive	5	Positive	R	Yes	NVL	0
Positive	**MSBTB MP434**	4	Positive	4	Positive	R	Yes	NVL	0
Positive	**MSBTB MP435**	0	Negative	0	Negative	R	Yes	NVL	0
Positive	**MSBTB MP436**	6	Positive	5	Positive	R	Yes	NVL	0
Positive	**MSBTB MP437**	4	Positive	4	Positive	R	Yes	NVL	0
Positive	**MSBTB MP438**	5	Positive	5	Positive	R	Yes	NVL	0
Positive	**MSBTB MP439**	7	Positive	5	Positive	R	Yes	NVL	0
Positive	**MSBTB MP440**	2	Positive	2	Positive	R	Yes	NVL	0
Positive	**MSBTB MP441**	5	Positive	5	Positive	R	Yes	NVL	0
Positive	**MSBTB MP442**	2	Positive	1	Negative	R	Yes	NVL	0
Positive	**MSBTB MP443**	4	Positive	4	Positive	R	Yes	NVL	0
Positive	**MSBTB MP444**	5	Positive	4	Positive	R	Yes	NVL	0
Positive	**MSBTB MP445**	3	Positive	3	Positive	R	Yes	NVL	0
Positive	**MSBTB MP450**	8	Positive	8	Positive	R	Yes	NVL	0
Positive	**MSBTB MP451 Repeat**	6	Positive	6	Positive	R	Yes	NVL	0
Positive	**MSBTB SP400**	0	Negative	0	Negative	R	Yes	NVL	0
Positive	**MSBTB SP449**	3	Positive	3	Positive	R	Yes	NVL	0
Positive	**MSBTB SP449 Repeat 10.9.21**	3	Positive	3	Positive	R	Yes	No PM	
Positive	**BTB2810200002**	4	Positive	3	Positive	R	Yes	No PM	
Positive	**BTB2810200026**	6	Positive	4	Positive	R	Yes	No PM	
Positive	**BTB2810200055**	4	Positive	2	Positive	R	Yes	No PM	
Positive	**BTB2810200079**	6	Positive	6	Positive	R	Yes	No PM	
Positive	**BTB2810200170**	0	Negative	0	Negative	R	Yes	No PM	
Positive	**MSBTB MP458**	7	Positive	7	Positive	**R**	Yes	No PM	
Positive	**MSBTB MP466**	1	Negative	1	Negative	**R**	Yes	No PM	
Positive	**MSBTB MP468**	7	Positive	5	Positive	R	Yes	VL	1

Positive	**MSBTB MP469**	5	Positive	4	Positive	R	Yes	No PM	
Positive	**MSBTB MP470**	7	Positive	6	Positive	R	Yes	No PM	
Positive	**MSBTB MP471**	5	Positive	5	Positive	R	Yes	No PM	
Positive	**MSBTB MP472**	7	Positive	5	Positive	R	Yes	No PM	
Positive	**MSBTB MP492**	5	Positive	5	Positive	R	Yes	NVL	0
Positive	**MSBTB MP503**	5	Positive	5	Positive	R	Yes	NVL	0
Positive	**MSBTB MP506**	5	Positive	5	Positive	R	Yes	NVL	0
Positive	**MSBTB MP508**	1	Negative	0	Negative	R	Yes	NVL	0
Positive	**MSBTB MP512**	10	Positive	9	Positive	R	Yes	VL	N/A
Positive	**MSBTB MP515**	2	Positive	2	Positive	2xIR	Yes	VL	N/A
Positive	**MSBTB MP517**	2	Positive	2	Positive	R	Yes	NVL	0
Positive	**MSBTB MP518**	1	Negative	1	Negative	R	Yes	NVL	0
Positive	**MSBTB MP519**	7	Positive	4	Positive	R	Yes	NVL	0
Positive	**MSBTB MP521**	0	Negative	0	Negative	R	Yes	NVL	0
Positive	**MSBTB MP522**	1	Negative	1	Negative	R	Yes	VL	multiple
Positive	**MSBTB MP523**	5	Positive	3	Positive	R	Yes	NVL	0
Positive	**MSBTB MP524**	9	Positive	10	Positive	R	Yes	VL	multiple
Positive	**MSBTB MP526**	6	Positive	5	Positive	R	Yes	NVL	0
Positive	**MSBTB MP528**	1	Negative	0	Negative	R	Yes	NVL	0
Positive	**MSBTB MP529**	4	Positive	2	Positive	R	Yes	NVL	0
Positive	**MSBTB MP531**	9	Positive	9	Positive	R	Yes	NVL	0
Positive	**MSBTB MP532**	0	Negative	0	Negative	R	Yes	NVL	0
Positive	**MSBTB MP533**	3	Positive	3	Positive	R	Yes	NVL	0
Positive	**MSBTB MP534**	5	Positive	5	Positive	R	Yes	NVL	0
Positive	**MSBTB MP535**	8	Positive	8	Positive	R	Yes	VL	0
Positive	**MSBTB MP538**	8	Positive	8	Positive	R	Yes	NVL	0
Positive	**MSBTB MP567**	4	Positive	4	Positive	R	Yes	NVL	0
Positive	**MSBTB MP568**	2	Positive	2	Positive	R	Yes	NVL	0
Positive	**MSBTB MP569**	5	Positive	4	Positive	R	Yes	NVL	0
Positive	**MSBTB MP570**	7	Positive	6	Positive	R	Yes	NVL	0
Positive	**MSBTB MP572**	10	Positive	10	Positive	R	Yes	NVL	0

Positive	**MSBTB MP573**	3	Positive	2	Positive	R	Yes	NVL	0
Positive	**MSBTB MP574**	7	Positive	7	Positive	R	Yes	NVL	0
Positive	**MSBTB MP575**	0	Negative	0	Negative	R	Yes	NVL	0
Positive	**MSBTB MP576**	5	Positive	5	Positive	R	Yes	NVL	0
Positive	**MSBTB MP577**	6	Positive	5	Positive	R	Yes	NVL	0
Positive	**MSBTB MP578**	4	Positive	4	Positive	R	Yes	NVL	0
Positive	**MSBTB MP579**	10	Positive	9	Positive	R	Yes	NVL	0
Positive	**MSBTB MP581**	8	Positive	8	Positive	R	Yes	NVL	0
Positive	**MSBTB MP586**	6	Positive	5	Positive	R	Yes	No PM	
Positive	**MSBTB MP587**	10	Positive	8	Positive	R	Yes	VL	1
Positive	**MSBTB MP592**	10	Positive	10	Positive	R	Yes	VL	1
Positive	**MSBTB MP593**	6	Positive	6	Positive	R	Yes	No PM	
Positive	**MSBTB MP594**	1	Negative	0	Negative	2xIR	Yes	No PM	
Positive	**MSBTB MP595**	1	Negative	1	Negative	2xIR	Yes	No PM	
Positive	**MSBTB MP597**	2	Positive	1	Negative	2xIR	Yes	No PM	
Positive	**MSBTB MP607**	6	Positive	5	Positive	2xIR	Yes	VL	1
Positive	**MSBTB MP610**	5	Positive	5	Positive	R	Yes	No PM	
Positive	**MSBTB MP612**	1	Negative	0	Negative	R	Yes	No PM	
Positive	**MSBTB MP616**	8	Positive	6	Positive	R	Yes	VL	multiple
Positive	**MSBTB MP617**	7	Positive	5	Positive	R	Yes	No PM	
Positive	**MSBTB MP619**	9	Positive	9	Positive	R	Yes	No PM	
Positive	**MSBTB MP620**	3	Positive	2	Positive	R	Yes	NVL	0
Positive	**MSBTB MP621**	2	Positive	0	Negative	R	Yes	NVL	0
Positive	**MSBTB MP622**	5	Positive	4	Positive	R	Yes	NVL	0
Positive	**MSBTB MP623**	6	Positive	6	Positive	R	Yes	NVL	0
Positive	**MSBTB MP624**	0	Negative	0	Negative	R	Yes	NVL	0
Positive	**MSBTB MP625**	6	Positive	4	Positive	R	Yes	NVL	0
Positive	**MSBTB MP626**	0	Negative	0	Negative	R	Yes	NVL	0
Positive	**MSBTB MP627**	6	Positive	6	Positive	R	Yes	NVL	0
Positive	**MSBTB MP628**	6	Positive	5	Positive	R	Yes	NVL	0
Positive	**MSBTB MP629**	7	Positive	7	Positive	R	Yes	NVL	0

Positive	**MSBTB MP630**	6	Positive	3	Positive	R	Yes	NVL	0
Positive	**MSBTB MP632**	5	Positive	5	Positive	2xIR	Yes	NVL	0
Positive	**MSBTB MP633**	6	Positive	5	Positive	R	Yes	NVL	0
Positive	**MSBTB MP634**	1	Negative	1	Negative	R	Yes	NVL	0
Positive	**MSBTB MP635**	3	Positive	3	Positive	R	Yes	VL	1
Positive	**MSBTB MP637**	4	Positive	4	Positive	R	Yes	NVL	0
Positive	**MSBTB MP638**	5	Positive	4	Positive	R	Yes	NVL	0
Positive	**MSBTB MP639**	7	Positive	6	Positive	R	Yes	NVL	0
Positive	**MSBTB MP644**	6	Positive	5	Positive	R	Yes	NVL	0
Positive	**MSBTB MP653**	6	Positive	5	Positive	R	Yes	VL	N/A
Positive	**MSBTB MP654**	9	Positive	9	Positive	R	Yes	VL	N/A
Positive	**MSBTB MP656**	10	Positive	9	Positive	R	Yes	VL	N/A
Positive	**MSBTB MP658**	10	Positive	9	Positive	R	Yes	VL	N/A
Positive	**MSBTB MP660**	6	Positive	6	Positive	R	Yes	VL	N/A
Positive	**MSBTB MP663**	9	Positive	7	Positive	R	Yes	NVL	0
Positive	**MSBTB MP664**	5	Positive	5	Positive	R	Yes	NVL	0
Positive	**MSBTB MP672**	9	Positive	8	Positive	2xIR	Yes	NVL	0
Positive	**MSBTB MP677**	9	Positive	9	Positive	2xIR	Yes	NVL	0
Positive	**MSBTB MP678**	6	Positive	6	Positive	R	Yes	NVL	0
Positive	**MSBTB MP686**	6	Positive	5	Positive	R	Yes	NVL	0
Positive	**MSBTB MP688**	6	Positive	6	Positive	R	Yes	NVL	0
Positive	**MSBTB MP689**	5	Positive	3	Positive	R	Yes	NVL	0
Positive	**MSBTB MP691**	3	Positive	3	Positive	R	Yes	NVL	0
Positive	**MSBTB MP692**	6	Positive	5	Positive	R	Yes	NVL	0
Positive	**MSBTB MP695**	3	Positive	4	Positive	R	Yes	NVL	0
Positive	**MSBTB MP735**	2	Positive	0	Negative	R	Yes	NVL	0
Positive	**MSBTB MP747**	5	Positive	5	Positive	R	Yes	NVL	0
Positive	**MSBTB MP748**	8	Positive	6	Positive	R	Yes	VL	multiple
Positive	**MSBTB MP750**	6	Positive	5	Positive	R	Yes	VL	multiple
Positive	**MSBTB MP751**	7	Positive	6	Positive	R	Yes	NVL	0
Positive	**MSBTB MP753**	6	Positive	6	Positive	R	Yes	VL	multiple

Positive	**MSBTB MP758**	7	Positive	7	Positive	R	Yes	NVL	0
Positive	**MSBTB SP545**	10	Positive	8	Positive	R	Yes	NVL	0
Positive	**MSBTB SP546**	9	Positive	9	Positive	R	Yes	VL	multiple
Positive	**MSBTB SP548**	2	Positive	2	Positive	R	Yes	NVL	0
Positive	**MSBTB SP588**	4	Positive	3	Positive	R	Yes	No PM	
Positive	**MSBTB SP609**	5	Positive	2	Positive	R	Yes	No PM	
Positive	**MSBTB SP685**	8	Positive	7	Positive	R	Yes	NVL	0
Positive	**MSBTB SP694**	2	Positive	2	Positive	R	Yes	VL	0
Positive	**MSBTB SP730**	5	Positive	5	Positive	R	Yes	NVL	0
Negative	**UKBTB MN01**	0	Negative	0	Negative				
Negative	**UKBTB MN02**	0	Negative	0	Negative				
Negative	**UKBTB MN03**	0	Negative	0	Negative				
Negative	**UKBTB MN04**	0	Negative	0	Negative				
Negative	**UKBTB MN05**	0	Negative	0	Negative				
Negative	**UKBTB MN06**	0	Negative	0	Negative				
Negative	**UKBTB MN07**	0	Negative	0	Negative				
Negative	**UKBTB MN08**	0	Negative	0	Negative				
Negative	**UKBTB MN09**	0	Negative	0	Negative				
Negative	**UKBTB MN10**	0	Negative	0	Negative				
Negative	**UKBTB MN11**	0	Negative	0	Negative				
Negative	**UKBTB MN12**	0	Negative	0	Negative				
Negative	**UKBTB MN13**	0	Negative	0	Negative				
Negative	**UKBTB MN14**	0	Negative	0	Negative				
Negative	**UKBTB MN15**	0	Negative	0	Negative				
Negative	**UKBTB MN16**	0	Negative	0	Negative				
Negative	**UKBTB MN17**	0	Negative	0	Negative				
Negative	**UKBTB MN19**	0	Negative	0	Negative				
Negative	**UKBTB MN20**	0	Negative	0	Negative				
Negative	**UKBTB MN21**	0	Negative	0	Negative				
Negative	**UKBTB MN22**	0	Negative	0	Negative				
Negative	**UKBTB MN23**	0	Negative	0	Negative				

Negative	**UKBTB MN24**	0	Negative	0	Negative
Negative	**UKBTB MN25**	0	Negative	0	Negative
Negative	**UKBTB MN26**	0	Negative	0	Negative
Negative	**UKBTB MN27**	0	Negative	0	Negative
Negative	**UKBTB MN28**	0	Negative	0	Negative
Negative	**UKBTB MN29**	0	Negative	0	Negative
Negative	**UKBTB MN30**	0	Negative	0	Negative
Negative	**UKBTB MN31**	0	Negative	0	Negative
Negative	**UKBTB MN32**	0	Negative	0	Negative
Negative	**UKBTB MN33**	0	Negative	0	Negative
Negative	**UKBTB MN34**	1	Negative	0	Negative
Negative	**UKBTB MN35**	0	Negative	0	Negative
Negative	**UKBTB MN36**	0	Negative	0	Negative
Negative	**UKBTB MN37**	0	Negative	0	Negative
Negative	**UKBTB MN38**	0	Negative	0	Negative
Negative	**UKBTB MN39**	0	Negative	0	Negative
Negative	**UKBTB MN40**	0	Negative	0	Negative
Negative	**UKBTB MN41**	0	Negative	0	Negative
Negative	**UKBTB MN42**	1	Negative	0	Negative
Negative	**UKBTB MN43**	0	Negative	0	Negative
Negative	**UKBTB MN44**	0	Negative	0	Negative
Negative	**UKBTB MN45**	0	Negative	0	Negative
Negative	**UKBTB MN46**	0	Negative	0	Negative
Negative	**UKBTB MN47**	0	Negative	0	Negative
Negative	**UKBTB MN48**	0	Negative	0	Negative
Negative	**UKBTB MN49**	0	Negative	0	Negative
Negative	**UKBTB MN50**	0	Negative	0	Negative
Negative	**UKBTB MN51**	0	Negative	0	Negative
Negative	**UKBTB MN52**	0	Negative	0	Negative
Negative	**UKBTB MN53**	0	Negative	0	Negative
Negative	**UKBTB MN54**	0	Negative	0	Negative

Negative	**UKBTB MN55**	0	Negative	0	Negative
Negative	**UKBTB MN56**	0	Negative	0	Negative
Negative	**UKBTB MN57**	0	Negative	0	Negative
Negative	**UKBTB MN58**	0	Negative	0	Negative
Negative	**UKBTB MN59**	0	Negative	0	Negative
Negative	**UKBTB MN60**	0	Negative	0	Negative
Negative	**UKBTB MN61**	0	Negative	0	Negative
Negative	**UKBTB MN62**	0	Negative	0	Negative
Negative	**UKBTB MN63**	0	Negative	0	Negative
Negative	**UKBTB MN64**	0	Negative	0	Negative
Negative	**UKBTB MN65**	0	Negative	0	Negative
Negative	**UKBTB MN66**	0	Negative	0	Negative
Negative	**UKBTB MN67**	0	Negative	0	Negative
Negative	**UKBTB MN68**	0	Negative	0	Negative
Negative	**UKBTB MN69**	0	Negative	0	Negative
Negative	**UKBTB MN70**	0	Negative	0	Negative
Negative	**UKBTB MN71**	0	Negative	0	Negative
Negative	**UKBTB MN72**	0	Negative	0	Negative
Negative	**UKBTB MN73**	0	Negative	0	Negative
Negative	**UKBTB MN74**	1	Negative	0	Negative
Negative	**UKBTB MN75**	0	Negative	0	Negative
Negative	**UKBTB MN76**	0	Negative	0	Negative
Negative	**UKBTB MN77**	0	Negative	0	Negative
Negative	**UKBTB MN78**	0	Negative	0	Negative
Negative	**UKBTB MN79**	0	Negative	0	Negative
Negative	**UKBTB MN80**	0	Negative	0	Negative
Negative	**UKBTB MN81**	0	Negative	0	Negative
Negative	**UKBTB MN82**	0	Negative	0	Negative
Negative	**UKBTB MN83**	0	Negative	0	Negative
Negative	**UKBTB MN84**	0	Negative	0	Negative
Negative	**UKBTB MN85**	0	Negative	0	Negative

Negative	**UKBTB MN86**	0	Negative	0	Negative
Negative	**UKBTB MN87**	0	Negative	0	Negative
Negative	**UKBTB MN88**	0	Negative	0	Negative
Negative	**UKBTB MN89**	0	Negative	0	Negative
Negative	**UKBTB MN90**	1	Negative	0	Negative
Negative	**UKBTB MN91**	0	Negative	0	Negative
Negative	**UKBTB MN92**	0	Negative	0	Negative
Negative	**UKBTB MN93**	0	Negative	0	Negative
Negative	**UKBTB MN94**	0	Negative	0	Negative
Negative	**UKBTB MN95**	0	Negative	0	Negative
Negative	**UKBTB MN96**	0	Negative	0	Negative
Negative	**UKBTB MN97**	1	Negative	0	Negative
Negative	**UKBTB MN98**	0	Negative	0	Negative
Negative	**UKBTB MN99**	0	Negative	0	Negative
Negative	**UKBTB MN100**	0	Negative	0	Negative
Negative	**UKBTB MN101**	0	Negative	0	Negative
Negative	**UKBTB MN102**	0	Negative	0	Negative
Negative	**UKBTB MN103**	0	Negative	0	Negative
Negative	**UKBTB MN104**	0	Negative	0	Negative
Negative	**UKBTB MN105**	0	Negative	0	Negative
Negative	**UKBTB MN106**	0	Negative	0	Negative
Negative	**UKBTB MN107**	0	Negative	0	Negative
Negative	**UKBTB MN108**	1	Negative	0	Negative
Negative	**UKBTB MN109**	0	Negative	0	Negative
Negative	**UKBTB MN110**	0	Negative	0	Negative
Negative	**UKBTB MN111**	0	Negative	0	Negative
Negative	**UKBTB MN112**	0	Negative	0	Negative
Negative	**UKBTB MN113**	0	Negative	0	Negative
Negative	**UKBTB MN114**	0	Negative	0	Negative
Negative	**UKBTB MN115**	0	Negative	0	Negative
Negative	**UKBTB MN116**	0	Negative	0	Negative

Negative	**UKBTB MN117**	0	Negative	0	Negative
Negative	**UKBTB MN118**	0	Negative	0	Negative
Negative	**UKBTB MN119**	0	Negative	0	Negative
Negative	**UKBTB MN120**	0	Negative	0	Negative
Negative	**UKBTB MN121**	0	Negative	0	Negative
Negative	**UKBTB MN122**	0	Negative	0	Negative
Negative	**UKBTB MN123**	0	Negative	0	Negative
Negative	**UKBTB MN124**	0	Negative	0	Negative
Negative	**UKBTB MN125**	0	Negative	0	Negative
Negative	**UKBTB MN126**	0	Negative	0	Negative
Negative	**UKBTB MN127**	0	Negative	0	Negative
Negative	**UKBTB MN128**	0	Negative	0	Negative
Negative	**UKBTB MN129**	0	Negative	0	Negative
Negative	**UKBTB MN130**	0	Negative	0	Negative
Negative	**UKBTB MN131**	0	Negative	0	Negative
Negative	**UKBTB MN132**	0	Negative	0	Negative
Negative	**UKBTB MN133**	0	Negative	0	Negative
Negative	**UKBTB MN134**	0	Negative	0	Negative
Negative	**UKBTB MN135**	0	Negative	0	Negative
Negative	**UKBTB MN136**	0	Negative	0	Negative
Negative	**UKBTB MN137**	0	Negative	0	Negative
Negative	**UKBTB MN138**	0	Negative	0	Negative
Negative	**UKBTB MN139**	0	Negative	0	Negative
Negative	**UKBTB MN140**	0	Negative	0	Negative
Negative	**UKBTB MN141**	0	Negative	0	Negative
Negative	**UKBTB MN142**	0	Negative	0	Negative
Negative	**UKBTB MN143**	0	Negative	0	Negative
Negative	**UKBTB MN144**	0	Negative	0	Negative
Negative	**UKBTB MN145**	0	Negative	0	Negative
Negative	**UKBTB MN146**	0	Negative	0	Negative
Negative	**UKBTB MN147**	0	Negative	0	Negative

Negative	**UKBTB MN148**	0	Negative	0	Negative
Negative	**UKBTB MN149**	0	Negative	0	Negative
Negative	**UKBTB MN150**	0	Negative	0	Negative
Negative	**UKBTB MN151**	0	Negative	0	Negative
Negative	**UKBTB MN152**	0	Negative	0	Negative
Negative	**UKBTB MN153**	0	Negative	0	Negative
Negative	**UKBTB MN154**	0	Negative	0	Negative
Negative	**UKBTB MN155**	0	Negative	0	Negative
Negative	**UKBTB MN156**	0	Negative	0	Negative
Negative	**UKBTB MN157**	0	Negative	0	Negative
Negative	**UKBTB MN158**	0	Negative	0	Negative
Negative	**UKBTB MN159**	0	Negative	0	Negative
Negative	**UKBTB MN160**	0	Negative	0	Negative
Negative	**UKBTB MN161**	0	Negative	0	Negative
Negative	**UKBTB MN162**	0	Negative	0	Negative
Negative	**UKBTB MN163**	0	Negative	0	Negative
Negative	**UKBTB MN164**	0	Negative	0	Negative
Negative	**UKBTB MN165**	0	Negative	0	Negative
Negative	**UKBTB MN166**	0	Negative	0	Negative
Negative	**UKBTB MN167**	0	Negative	0	Negative
Negative	**UKBTB MN168**	0	Negative	0	Negative
Negative	**UKBTB MN169**	0	Negative	0	Negative
Negative	**UKBTB MN170**	0	Negative	0	Negative
Negative	**UKBTB MN171**	0	Negative	0	Negative
Negative	**UKBTB MN172**	0	Negative	0	Negative
Negative	**UKBTB MN173**	0	Negative	0	Negative
Negative	**UKBTB MN174**	0	Negative	0	Negative
Negative	**UKBTB MN175**	0	Negative	0	Negative
Negative	**UKBTB MN176**	0	Negative	0	Negative
Negative	**UKBTB MN177**	0	Negative	0	Negative
Negative	**UKBTB MN178**	0	Negative	0	Negative

Negative	**UKBTB MN179**	0	Negative	0	Negative
Negative	**UKBTB MN180**	0	Negative	0	Negative
Negative	**UKBTB MN181**	0	Negative	0	Negative
Negative	**UKBTB MN182**	0	Negative	0	Negative
Negative	**UKBTB MN183**	0	Negative	0	Negative
Negative	**UKBTB MN184**	0	Negative	0	Negative
Negative	**UKBTB MN185**	0	Negative	0	Negative
Negative	**UKBTB MN186**	0	Negative	0	Negative
Negative	**UKBTB MN187**	0	Negative	0	Negative
Negative	**UKBTB MN188**	0	Negative	0	Negative
Negative	**UKBTB MN189**	0	Negative	0	Negative
Negative	**UKBTB MN190**	0	Negative	0	Negative
Negative	**UKBTB MN191**	0	Negative	0	Negative
Negative	**UKBTB MN192**	0	Negative	0	Negative
Negative	**UKBTB MN193**	0	Negative	0	Negative
Negative	**UKBTB MN194**	1	Negative	0	Negative
Negative	**UKBTB MN195**	0	Negative	0	Negative
Negative	**UKBTB MN196**	1	Negative	0	Negative
Negative	**UKBTB MN197**	0	Negative	0	Negative
Negative	**UKBTB MN198**	0	Negative	0	Negative
Negative	**UKBTB MN199**	0	Negative	0	Negative
Negative	**UKBTB MN200**	0	Negative	0	Negative
Negative	**UKBTB MN201**	0	Negative	0	Negative
Negative	**UKBTB MN202**	0	Negative	0	Negative
Negative	**UKBTB MN203**	0	Negative	0	Negative
Negative	**UKBTB MN204**	0	Negative	0	Negative
Negative	**UKBTB MN205**	0	Negative	0	Negative
Negative	**UKBTB MN206**	0	Negative	0	Negative
Negative	**UKBTB MN207**	0	Negative	0	Negative
Negative	**UKBTB MN208**	0	Negative	0	Negative
Negative	**UKBTB MN209**	0	Negative	0	Negative

Negative	**UKBTB MN210**	0	Negative	0	Negative
Negative	**UKBTB MN211**	0	Negative	0	Negative
Negative	**UKBTB MN212**	0	Negative	0	Negative
Negative	**UKBTB MN213**	0	Negative	0	Negative
Negative	**UKBTB MN214**	0	Negative	0	Negative
Negative	**UKBTB MN215**	0	Negative	0	Negative
Negative	**UKBTB MN216**	0	Negative	0	Negative
Negative	**UKBTB MN217**	0	Negative	0	Negative
Negative	**UKBTB MN218**	0	Negative	0	Negative
Negative	**UKBTB MN219**	0	Negative	0	Negative
Negative	**UKBTB MN220**	0	Negative	0	Negative
Negative	**UKBTB MN221**	0	Negative	0	Negative
Negative	**UKBTB MN222**	0	Negative	0	Negative
Negative	**UKBTB MN223**	0	Negative	0	Negative
Negative	**UKBTB MN224**	0	Negative	0	Negative
Negative	**UKBTB MN225**	0	Negative	0	Negative
Negative	**UKBTB MN226**	0	Negative	0	Negative
Negative	**UKBTB MN227**	0	Negative	0	Negative
Negative	**UKBTB MN228**	0	Negative	0	Negative
Negative	**UKBTB MN229**	0	Negative	0	Negative
Negative	**UKBTB MN230**	0	Negative	0	Negative
Negative	**UKBTB MN231**	0	Negative	0	Negative
Negative	**UKBTB MN232**	0	Negative	0	Negative
Negative	**UKBTB MN233**	0	Negative	0	Negative
Negative	**UKBTB MN234**	0	Negative	0	Negative
Negative	**UKBTB MN235**	0	Negative	0	Negative
Negative	**UKBTB MN236**	0	Negative	0	Negative
Negative	**UKBTB MN237**	0	Negative	0	Negative
Negative	**UKBTB MN238**	0	Negative	0	Negative
Negative	**UKBTB MN239**	0	Negative	0	Negative
Negative	**UKBTB MN240**	0	Negative	0	Negative

Negative	**UKBTB MN241**	0	Negative	0	Negative
Negative	**UKBTB MN242**	0	Negative	0	Negative
Negative	**UKBTB MN243**	0	Negative	0	Negative
Negative	**UKBTB MN244**	0	Negative	0	Negative
Negative	**UKBTB MN245**	0	Negative	0	Negative
Negative	**UKBTB MN246**	0	Negative	0	Negative
Negative	**UKBTB MN247**	0	Negative	0	Negative
Negative	**UKBTB MN248**	0	Negative	0	Negative
Negative	**UKBTB MN249**	0	Negative	0	Negative
Negative	**UKBTB MN250**	0	Negative	0	Negative
Negative	**UKBTB MN251**	0	Negative	0	Negative
Negative	**UKBTB MN252**	0	Negative	0	Negative
Negative	**UKBTB MN253**	0	Negative	0	Negative
Negative	**UKBTB MN254**	0	Negative	0	Negative
Negative	**UKBTB MN255**	0	Negative	0	Negative
Negative	**UKBTB MN256**	0	Negative	0	Negative
Negative	**UKBTB MN257**	0	Negative	0	Negative
Negative	**UKBTB MN258**	0	Negative	0	Negative
Negative	**UKBTB MN259**	0	Negative	0	Negative
Negative	**UKBTB MN260**	0	Negative	0	Negative
Negative	**UKBTB MN261**	0	Negative	0	Negative
Negative	**UKBTB MN262**	0	Negative	0	Negative
Negative	**UKBTB MN263**	0	Negative	0	Negative
Negative	**UKBTB MN264**	0	Negative	0	Negative
Negative	**UKBTB MN265**	0	Negative	0	Negative
Negative	**UKBTB MN266**	1	Negative	0	Negative
Negative	**UKBTB MN267**	0	Negative	0	Negative
Negative	**UKBTB MN268**	0	Negative	0	Negative
Negative	**UKBTB MN269**	0	Negative	0	Negative
Negative	**UKBTB MN270**	0	Negative	0	Negative
Negative	**UKBTB MN271**	0	Negative	0	Negative

Negative	**UKBTB MN272**	0	Negative	0	Negative
Negative	**UKBTB MN273**	0	Negative	0	Negative
Negative	**UKBTB MN274**	1	Negative	0	Negative
Negative	**UKBTB MN275**	0	Negative	0	Negative
Negative	**UKBTB MN276**	0	Negative	0	Negative
Negative	**UKBTB MN277**	0	Negative	0	Negative
Negative	**UKBTB MN278**	0	Negative	0	Negative
Negative	**UKBTB MN279**	0	Negative	0	Negative
Negative	**UKBTB MN280**	0	Negative	0	Negative
Negative	**UKBTB MN281**	0	Negative	0	Negative
Negative	**UKBTB MN282**	1	Negative	0	Negative
Negative	**UKBTB MN283**	0	Negative	0	Negative
Negative	**UKBTB MN284**	0	Negative	0	Negative
Negative	**UKBTB MN285**	0	Negative	0	Negative
Negative	**UKBTB MN286**	0	Negative	0	Negative
Negative	**UKBTB MN287**	0	Negative	0	Negative
Negative	**UKBTB MN288**	0	Negative	0	Negative
Negative	**UKBTB MN289**	0	Negative	0	Negative
Negative	**UKBTB MN290**	0	Negative	0	Negative
Negative	**UKBTB MN291**	0	Negative	0	Negative
Negative	**UKBTB MN292**	0	Negative	0	Negative
Negative	**UKBTB MN293**	0	Negative	0	Negative
Negative	**UKBTB MN294**	0	Negative	0	Negative
Negative	**UKBTB MN295**	0	Negative	0	Negative
Negative	**UKBTB MN296**	0	Negative	0	Negative
Negative	**UKBTB MN297**	0	Negative	0	Negative
Negative	**UKBTB MN298**	0	Negative	0	Negative
Negative	**UKBTB MN299**	0	Negative	0	Negative
Negative	**UKBTB MN300**	0	Negative	0	Negative
Negative	**UKBTB MN301**	0	Negative	0	Negative
Negative	**UKBTB MN302**	0	Negative	0	Negative

Negative	**UKBTB MN303**	0	Negative	0	Negative
Negative	**UKBTB MN304**	0	Negative	0	Negative
Negative	**UKBTB MN305**	0	Negative	0	Negative
Negative	**UKBTB MN306**	0	Negative	0	Negative
Negative	**UKBTB MN307**	0	Negative	0	Negative
Negative	**UKBTB MN308**	0	Negative	0	Negative
Negative	**UKBTB MN309**	0	Negative	0	Negative
Negative	**UKBTB MN310**	0	Negative	0	Negative
Negative	**UKBTB MN311**	0	Negative	0	Negative
Negative	**UKBTB MN312**	0	Negative	0	Negative
Negative	**UKBTB MN313**	0	Negative	0	Negative
Negative	**UKBTB MN314**	0	Negative	0	Negative
Negative	**UKBTB MN315**	0	Negative	0	Negative
Negative	**UKBTB MN316**	0	Negative	0	Negative
Negative	**UKBTB MN317**	0	Negative	0	Negative
Negative	**UKBTB MN318**	0	Negative	0	Negative
Negative	**UKBTB MN319**	0	Negative	0	Negative
Negative	**UKBTB MN320**	0	Negative	0	Negative
Negative	**UKBTB MN321**	0	Negative	0	Negative
Negative	**UKBTB MN322**	0	Negative	0	Negative
Negative	**UKBTB MN323**	0	Negative	0	Negative
Negative	**UKBTB MN324**	0	Negative	0	Negative
Negative	**UKBTB MN325**	0	Negative	0	Negative
Negative	**UKBTB MN326**	0	Negative	0	Negative
Negative	**UKBTB MN327**	0	Negative	0	Negative
Negative	**UKBTB MN328**	0	Negative	0	Negative
Negative	**UKBTB MN329**	0	Negative	0	Negative
Negative	**UKBTB MN330**	0	Negative	0	Negative
Negative	**UKBTB MN331**	0	Negative	0	Negative
Negative	**UKBTB MN332**	0	Negative	0	Negative
Negative	**UKBTB MN333**	0	Negative	0	Negative

Negative	**UKBTB MN334**	0	Negative	0	Negative
Negative	**UKBTB MN335**	0	Negative	0	Negative
Negative	**UKBTB MN336**	0	Negative	0	Negative
Negative	**UKBTB MN337**	0	Negative	0	Negative
Negative	**UKBTB MN338**	0	Negative	0	Negative
Negative	**UKBTB MN339**	0	Negative	0	Negative
Negative	**UKBTB MN340**	0	Negative	0	Negative
Negative	**UKBTB MN341**	0	Negative	0	Negative
Negative	**UKBTB MN342**	0	Negative	0	Negative
Negative	**UKBTB MN343**	0	Negative	0	Negative
Negative	**UKBTB MN344**	0	Negative	0	Negative
Negative	**UKBTB MN345**	0	Negative	0	Negative
Negative	**UKBTB MN346**	0	Negative	0	Negative
Negative	**UKBTB MN347**	0	Negative	0	Negative
Negative	**UKBTB MN348**	0	Negative	0	Negative
Negative	**UKBTB MN349**	0	Negative	0	Negative
Negative	**UKBTB MN350**	0	Negative	0	Negative
Negative	**UKBTB MN351**	0	Negative	0	Negative
Negative	**UKBTB MN352**	0	Negative	0	Negative
Negative	**UKBTB MN353**	0	Negative	0	Negative
Negative	**UKBTB MN354**	0	Negative	0	Negative
Negative	**UKBTB MN355**	0	Negative	0	Negative
Negative	**UKBTB MN356**	0	Negative	0	Negative
Negative	**UKBTB MN357**	0	Negative	0	Negative
Negative	**UKBTB MN358**	0	Negative	0	Negative
Negative	**UKBTB MN359**	0	Negative	0	Negative
Negative	**UKBTB MN360**	0	Negative	0	Negative
Negative	**UKBTB MN361**	0	Negative	0	Negative
Negative	**UKBTB MN362**	0	Negative	0	Negative
Negative	**UKBTB MN363**	0	Negative	0	Negative
Negative	**UKBTB MN364**	0	Negative	0	Negative

Negative	**UKBTB MN365**	0	Negative	0	Negative
Negative	**UKBTB MN366**	0	Negative	0	Negative
Negative	**UKBTB MN367**	0	Negative	0	Negative
Negative	**UKBTB MN368**	0	Negative	0	Negative
Negative	**UKBTB MN369**	0	Negative	0	Negative
Negative	**UKBTB MN370**	0	Negative	0	Negative
Negative	**UKBTB MN371**	0	Negative	0	Negative
Negative	**UKBTB MN372**	0	Negative	0	Negative
Negative	**UKBTB MN373**	0	Negative	0	Negative
Negative	**UKBTB MN374**	0	Negative	0	Negative
Negative	**UKBTB MN375**	0	Negative	0	Negative
Negative	**UKBTB MN376**	0	Negative	0	Negative
Negative	**UKBTB MN377**	0	Negative	0	Negative
Negative	**UKBTB MN378**	0	Negative	0	Negative
Negative	**UKBTB MN379**	0	Negative	0	Negative
Negative	**UKBTB MN380**	0	Negative	0	Negative
Negative	**UKBTB MN381**	0	Negative	0	Negative
Negative	**UKBTB MN382**	0	Negative	0	Negative
Negative	**UKBTB MN383**	0	Negative	0	Negative
Negative	**UKBTB MN384**	0	Negative	0	Negative
Negative	**UKBTB MN385**	0	Negative	0	Negative
Negative	**UKBTB MN386**	0	Negative	0	Negative
Negative	**UKBTB MN387**	0	Negative	0	Negative
Negative	**UKBTB MN388**	0	Negative	0	Negative
Negative	**UKBTB MN389**	0	Negative	0	Negative
Negative	**UKBTB MN390**	0	Negative	0	Negative
Negative	**UKBTB MN391**	0	Negative	0	Negative
Negative	**UKBTB MN392**	0	Negative	0	Negative
Negative	**UKBTB MN393**	0	Negative	0	Negative
Negative	**UKBTB MN394**	0	Negative	0	Negative
Negative	**UKBTB MN395**	0	Negative	0	Negative

Negative	**UKBTB MN396**	0	Negative	0	Negative
Negative	**UKBTB MN397**	0	Negative	0	Negative
Negative	**UKBTB MN398**	0	Negative	0	Negative
Negative	**UKBTB MN399**	0	Negative	0	Negative
Negative	**UKBTB MN400**	0	Negative	0	Negative
Negative	**UKBTB MN401**	0	Negative	0	Negative
Negative	**UKBTB MN402**	0	Negative	0	Negative
Negative	**UKBTB MN403**	0	Negative	0	Negative
Negative	**UKBTB MN404**	0	Negative	0	Negative
Negative	**UKBTB MN405**	0	Negative	0	Negative
Negative	**UKBTB MN406**	0	Negative	0	Negative
Negative	**UKBTB MN407**	0	Negative	0	Negative
Negative	**UKBTB MN408**	0	Negative	0	Negative
Negative	**UKBTB MN409**	0	Negative	0	Negative
Negative	**UKBTB MN410**	0	Negative	0	Negative
Negative	**UKBTB MN411**	0	Negative	0	Negative
Negative	**UKBTB MN412**	0	Negative	0	Negative
Negative	**UKBTB MN413**	0	Negative	0	Negative
Negative	**UKBTB MN414**	0	Negative	0	Negative
Negative	**UKBTB MN415**	0	Negative	0	Negative
Negative	**UKBTB MN416**	0	Negative	0	Negative
Negative	**UKBTB MN417**	0	Negative	0	Negative
Negative	**UKBTB MN418**	0	Negative	0	Negative
Negative	**UKBTB MN419**	1	Negative	0	Negative
Negative	**UKBTB MN420**	0	Negative	0	Negative
Negative	**UKBTB MN421**	0	Negative	0	Negative
Negative	**UKBTB MN422**	0	Negative	0	Negative
Negative	**UKBTB MN423**	0	Negative	0	Negative
Negative	**UKBTB MN424**	0	Negative	0	Negative
Negative	**UKBTB MN425**	0	Negative	0	Negative
Negative	**UKBTB MN426**	0	Negative	0	Negative

Negative	**UKBTB MN427**	0	Negative	0	Negative
Negative	**UKBTB MN428**	0	Negative	0	Negative
Negative	**UKBTB MN429**	0	Negative	0	Negative
Negative	**UKBTB MN430**	0	Negative	0	Negative
Negative	**UKBTB MN431**	0	Negative	0	Negative
Negative	**UKBTB MN432**	0	Negative	0	Negative
Negative	**UKBTB MN433**	0	Negative	0	Negative
Negative	**UKBTB MN434**	0	Negative	0	Negative
Negative	**UKBTB MN435**	0	Negative	0	Negative
Negative	**UKBTB MN436**	0	Negative	0	Negative
Negative	**UKBTB MN437**	0	Negative	0	Negative
Negative	**UKBTB MN438**	1	Negative	0	Negative
Negative	**UKBTB MN439**	0	Negative	0	Negative
Negative	**UKBTB MN440**	0	Negative	0	Negative
Negative	**UKBTB MN441**	0	Negative	0	Negative
Negative	**UKBTB MN442**	0	Negative	0	Negative
Negative	**UKBTB MN443**	0	Negative	0	Negative
Negative	**UKBTB MN444**	0	Negative	0	Negative
Negative	**UKBTB MN445**	0	Negative	0	Negative
Negative	**UKBTB MN446**	0	Negative	0	Negative
Negative	**UKBTB MN447**	0	Negative	0	Negative
Negative	**UKBTB MN448**	0	Negative	0	Negative
Negative	**UKBTB MN449**	0	Negative	0	Negative
Negative	**UKBTB MN450**	0	Negative	0	Negative
Negative	**UKBTB MN451**	0	Negative	0	Negative
Negative	**UKBTB MN452**	0	Negative	0	Negative
Negative	**UKBTB MN453**	0	Negative	0	Negative
Negative	**UKBTB MN454**	0	Negative	0	Negative
Negative	**UKBTB MN455**	0	Negative	0	Negative
Negative	**UKBTB MN456**	0	Negative	0	Negative
Negative	**UKBTB MN457**	0	Negative	0	Negative

Negative	**UKBTB MN458**	0	Negative	0	Negative
Negative	**UKBTB MN459**	0	Negative	0	Negative
Negative	**UKBTB MN460**	0	Negative	0	Negative
Negative	**UKBTB MN461**	0	Negative	0	Negative
Negative	**UKBTB MN462**	0	Negative	0	Negative
Negative	**UKBTB MN463**	0	Negative	0	Negative
Negative	**UKBTB MN464**	0	Negative	0	Negative
Negative	**UKBTB MN465**	0	Negative	0	Negative
Negative	**UKBTB MN466**	0	Negative	0	Negative
Negative	**UKBTB MN467**	0	Negative	0	Negative
Negative	**UKBTB MN468**	0	Negative	0	Negative
Negative	**UKBTB MN469**	0	Negative	0	Negative
Negative	**UKBTB MN470**	0	Negative	0	Negative
Negative	**UKBTB MN471**	0	Negative	0	Negative
Negative	**UKBTB MN472**	0	Negative	0	Negative
Negative	**UKBTB MN473**	0	Negative	0	Negative
Negative	**UKBTB MN474**	0	Negative	0	Negative
Negative	**UKBTB MN475**	0	Negative	0	Negative
Negative	**UKBTB MN476**	0	Negative	0	Negative
Negative	**UKBTB MN477**	0	Negative	0	Negative
Negative	**UKBTB MN478**	0	Negative	0	Negative
Negative	**UKBTB MN479**	0	Negative	0	Negative
Negative	**UKBTB MN480**	0	Negative	0	Negative
Negative	**UKBTB MN481**	0	Negative	0	Negative
Negative	**UKBTB MN482**	0	Negative	0	Negative
Negative	**UKBTB MN483**	0	Negative	0	Negative
Negative	**UKBTB MN484**	0	Negative	0	Negative
Negative	**UKBTB MN485**	0	Negative	0	Negative
Negative	**UKBTB MN486**	0	Negative	0	Negative
Negative	**UKBTB MN487**	0	Negative	0	Negative
Negative	**UKBTB MN488**	0	Negative	0	Negative

Negative	**UKBTB MN489**	0	Negative	0	Negative
Negative	**UKBTB MN490**	0	Negative	0	Negative
Negative	**UKBTB MN491**	0	Negative	0	Negative
Negative	**UKBTB MN492**	0	Negative	0	Negative
Negative	**UKBTB MN493**	0	Negative	0	Negative
Negative	**UKBTB MN494**	0	Negative	0	Negative
Negative	**UKBTB MN495**	0	Negative	0	Negative
Negative	**UKBTB MN496**	0	Negative	0	Negative
Negative	**UKBTB MN497**	0	Negative	0	Negative
Negative	**UKBTB MN498**	0	Negative	0	Negative
Negative	**UKBTB MN499**	0	Negative	0	Negative
Negative	**UKBTB MN500**	0	Negative	0	Negative
Negative	**UKBTB MN501**	0	Negative	0	Negative
Negative	**UKBTB MN502**	0	Negative	0	Negative
Negative	**UKBTB MN503**	0	Negative	0	Negative
Negative	**UKBTB MN504**	0	Negative	0	Negative
Negative	**UKBTB MN505**	0	Negative	0	Negative
Negative	**UKBTB MN506**	0	Negative	0	Negative
Negative	**UKBTB MN507**	0	Negative	0	Negative
Negative	**UKBTB MN508**	0	Negative	0	Negative
Negative	**UKBTB MN509**	0	Negative	0	Negative
Negative	**UKBTB MN510**	0	Negative	0	Negative
Negative	**UKBTB MN511**	0	Negative	0	Negative
Negative	**UKBTB MN512**	0	Negative	0	Negative
Negative	**UKBTB MN513**	0	Negative	0	Negative
Negative	**UKBTB MN514**	0	Negative	0	Negative
Negative	**UKBTB MN515**	0	Negative	0	Negative
Negative	**UKBTB MN516**	0	Negative	0	Negative
Negative	**UKBTB MN517**	0	Negative	0	Negative
Negative	**UKBTB MN518**	0	Negative	0	Negative
Negative	**UKBTB MN519**	0	Negative	0	Negative

Negative	**UKBTB MN520**	0	Negative	0	Negative
Negative	**UKBTB MN521**	0	Negative	0	Negative
Negative	**UKBTB MN522**	0	Negative	0	Negative
Negative	**UKBTB MN523**	0	Negative	0	Negative
Negative	**UKBTB MN524**	0	Negative	0	Negative
Negative	**UKBTB MN525**	0	Negative	0	Negative
Negative	**UKBTB MN526**	0	Negative	0	Negative
Negative	**UKBTB MN527**	0	Negative	0	Negative
Negative	**UKBTB MN528**	0	Negative	0	Negative
Negative	**UKBTB MN529**	0	Negative	0	Negative
Negative	**UKBTB MN530**	0	Negative	0	Negative
Negative	**UKBTB MN531**	0	Negative	0	Negative
Negative	**UKBTB MN532**	0	Negative	0	Negative
Negative	**UKBTB MN533**	0	Negative	0	Negative
Negative	**UKBTB MN534**	0	Negative	0	Negative
Negative	**UKBTB MN535**	0	Negative	0	Negative
Negative	**UKBTB MN536**	0	Negative	0	Negative
Negative	**UKBTB MN537**	0	Negative	0	Negative
Negative	**UKBTB MN538**	0	Negative	0	Negative
Negative	**UKBTB MN539**	0	Negative	0	Negative
Negative	**UKBTB MN540**	0	Negative	0	Negative
Negative	**UKBTB MN541**	0	Negative	0	Negative
Negative	**UKBTB MN542**	0	Negative	0	Negative
Negative	**UKBTB MN543**	0	Negative	0	Negative
Negative	**UKBTB MN544**	0	Negative	0	Negative
Negative	**UKBTB MN545**	0	Negative	0	Negative
Negative	**UKBTB MN546**	0	Negative	0	Negative
Negative	**UKBTB MN547**	0	Negative	0	Negative
Negative	**UKBTB MN548**	0	Negative	0	Negative
Negative	**UKBTB MN549**	0	Negative	0	Negative
Negative	**UKBTB MN550**	0	Negative	0	Negative

Negative	**UKBTB MN551**	0	Negative	0	Negative
Negative	**UKBTB MN552**	0	Negative	0	Negative
Negative	**UKBTB MN553**	0	Negative	0	Negative
Negative	**UKBTB MN554**	0	Negative	0	Negative
Negative	**UKBTB MN555**	0	Negative	0	Negative
Negative	**UKBTB MN556**	0	Negative	0	Negative
Negative	**UKBTB MN557**	0	Negative	0	Negative
Negative	**UKBTB MN558**	0	Negative	0	Negative
Negative	**UKBTB MN559**	0	Negative	0	Negative
Negative	**UKBTB MN560**	0	Negative	0	Negative
Negative	**UKBTB MN561**	0	Negative	0	Negative
Negative	**UKBTB MN562**	0	Negative	0	Negative
Negative	**UKBTB MN563**	0	Negative	0	Negative
Negative	**UKBTB MN564**	0	Negative	0	Negative
Negative	**UKBTB MN565**	0	Negative	0	Negative
Negative	**UKBTB MN566**	0	Negative	0	Negative
Negative	**UKBTB MN567**	0	Negative	0	Negative
Negative	**UKBTB MN568**	0	Negative	0	Negative
Negative	**UKBTB MN569**	0	Negative	0	Negative
Negative	**UKBTB MN570**	0	Negative	0	Negative
Negative	**UKBTB MN571**	1	Negative	0	Negative
Negative	**UKBTB MN572**	0	Negative	0	Negative
Negative	**UKBTB MN573**	0	Negative	0	Negative
Negative	**UKBTB MN574**	0	Negative	0	Negative
Negative	**UKBTB MN575**	0	Negative	0	Negative
Negative	**UKBTB MN576**	0	Negative	0	Negative
Negative	**UKBTB MN577**	0	Negative	0	Negative
Negative	**UKBTB MN578**	0	Negative	0	Negative
Negative	**UKBTB MN579**	0	Negative	0	Negative
Negative	**UKBTB MN580**	0	Negative	0	Negative
Negative	**UKBTB MN581**	0	Negative	0	Negative

Negative	**UKBTB MN582**	0	Negative	0	Negative
Negative	**UKBTB MN583**	0	Negative	0	Negative
Negative	**UKBTB MN584**	0	Negative	0	Negative
Negative	**UKBTB MN585**	0	Negative	0	Negative
Negative	**UKBTB MN586**	0	Negative	0	Negative
Negative	**UKBTB MN587**	0	Negative	0	Negative
Negative	**UKBTB MN588**	0	Negative	0	Negative
Negative	**UKBTB MN589**	0	Negative	0	Negative
Negative	**UKBTB MN590**	0	Negative	0	Negative
Negative	**UKBTB MN591**	0	Negative	0	Negative
Negative	**UKBTB MN592**	0	Negative	0	Negative
Negative	**UKBTB MN593**	0	Negative	0	Negative
Negative	**UKBTB MN594**	0	Negative	0	Negative
Negative	**UKBTB MN595**	0	Negative	0	Negative
Negative	**UKBTB MN596**	0	Negative	0	Negative
Negative	**UKBTB MN597**	0	Negative	0	Negative
Negative	**UKBTB MN598**	0	Negative	0	Negative
Negative	**UKBTB MN599**	0	Negative	0	Negative
Negative	**UKBTB MN600**	0	Negative	0	Negative
Negative	**UKBTB MN601**	0	Negative	0	Negative
Negative	**UKBTB MN602**	0	Negative	0	Negative
Negative	**UKBTB MN603**	0	Negative	0	Negative
Negative	**UKBTB MN604**	0	Negative	0	Negative
Negative	**UKBTB MN605**	0	Negative	0	Negative
Negative	**UKBTB MN606**	0	Negative	0	Negative
Negative	**UKBTB MN607**	0	Negative	0	Negative
Negative	**UKBTB MN608**	0	Negative	0	Negative
Negative	**UKBTB MN609**	0	Negative	0	Negative
Negative	**UKBTB MN610**	0	Negative	0	Negative
Negative	**UKBTB MN611**	0	Negative	0	Negative
Negative	**UKBTB MN612**	0	Negative	0	Negative

Negative	**UKBTB MN613**	0	Negative	0	Negative
Negative	**UKBTB MN614**	0	Negative	0	Negative
Negative	**UKBTB MN615**	0	Negative	0	Negative
Negative	**UKBTB MN616**	0	Negative	0	Negative
Negative	**UKBTB MN617**	0	Negative	0	Negative
Negative	**UKBTB MN618**	1	Negative	0	Negative
Negative	**UKBTB MN619**	0	Negative	0	Negative
Negative	**UKBTB MN620**	0	Negative	0	Negative
Negative	**UKBTB MN621**	0	Negative	0	Negative
Negative	**UKBTB MN622**	0	Negative	0	Negative
Negative	**UKBTB MN623**	0	Negative	0	Negative
Negative	**UKBTB MN624**	0	Negative	0	Negative
Negative	**UKBTB MN625**	0	Negative	0	Negative
Negative	**UKBTB MN626**	0	Negative	0	Negative
Negative	**UKBTB MN627**	0	Negative	0	Negative
Negative	**UKBTB MN628**	0	Negative	0	Negative
Negative	**UKBTB MN629**	0	Negative	0	Negative
Negative	**UKBTB MN630**	0	Negative	0	Negative
Negative	**UKBTB MN631**	0	Negative	0	Negative
Negative	**UKBTB MN632**	0	Negative	0	Negative
Negative	**UKBTB MN633**	0	Negative	0	Negative
Negative	**UKBTB MN634**	0	Negative	0	Negative
Negative	**UKBTB MN635**	0	Negative	0	Negative
Negative	**UKBTB MN636**	0	Negative	0	Negative
Negative	**UKBTB MN637**	0	Negative	0	Negative
Negative	**UKBTB MN638**	0	Negative	0	Negative
Negative	**UKBTB MN639**	0	Negative	0	Negative
Negative	**UKBTB MN640**	0	Negative	0	Negative
Negative	**UKBTB MN641**	0	Negative	0	Negative
Negative	**UKBTB MN642**	0	Negative	0	Negative
Negative	**UKBTB MN643**	0	Negative	0	Negative

Negative	**UKBTB MN644**	0	Negative	0	Negative
Negative	**UKBTB MN645**	1	Negative	1	Negative
Negative	**UKBTB MN646**	0	Negative	0	Negative
Negative	**UKBTB MN647**	0	Negative	0	Negative
Negative	**UKBTB MN648**	0	Negative	0	Negative
Negative	**UKBTB MN649**	0	Negative	0	Negative
Negative	**UKBTB MN650**	0	Negative	0	Negative
Negative	**UKBTB MN651**	0	Negative	0	Negative
Negative	**UKBTB MN652**	0	Negative	0	Negative
Negative	**UKBTB MN653**	0	Negative	0	Negative
Negative	**UKBTB MN654**	0	Negative	0	Negative
Negative	**UKBTB MN655**	0	Negative	0	Negative
Negative	**UKBTB MN656**	0	Negative	0	Negative
Negative	**UKBTB MN657**	0	Negative	0	Negative
Negative	**UKBTB MN658**	0	Negative	0	Negative
Negative	**UKBTB MN659**	0	Negative	0	Negative
Negative	**UKBTB MN660**	0	Negative	0	Negative
Negative	**UKBTB MN661**	0	Negative	0	Negative
Negative	**UKBTB MN662**	0	Negative	0	Negative
Negative	**UKBTB MN663**	0	Negative	0	Negative
Negative	**UKBTB MN664**	0	Negative	0	Negative
Negative	**UKBTB MN665**	0	Negative	0	Negative
Negative	**UKBTB MN666**	0	Negative	0	Negative
Negative	**UKBTB MN667**	0	Negative	0	Negative
Negative	**UKBTB MN668**	0	Negative	0	Negative
Negative	**UKBTB MN669**	0	Negative	0	Negative
Negative	**UKBTB MN670**	0	Negative	0	Negative
Negative	**UKBTB MN671**	0	Negative	0	Negative
Negative	**UKBTB MN672**	0	Negative	0	Negative
Negative	**UKBTB MN673**	0	Negative	0	Negative
Negative	**UKBTB MN674**	0	Negative	0	Negative

Negative	**UKBTB MN675**	0	Negative	0	Negative
Negative	**UKBTB MN676**	0	Negative	0	Negative
Negative	**UKBTB MN677**	0	Negative	0	Negative
Negative	**UKBTB MN678**	1	Negative	1	Negative
Negative	**UKBTB MN679**	0	Negative	0	Negative
Negative	**UKBTB MN680**	0	Negative	0	Negative
Negative	**UKBTB MN681**	0	Negative	0	Negative
Negative	**UKBTB MN682**	0	Negative	0	Negative
Negative	**UKBTB MN683**	0	Negative	0	Negative
Negative	**UKBTB MN684**	0	Negative	0	Negative
Negative	**UKBTB MN685**	0	Negative	0	Negative
Negative	**UKBTB MN686**	0	Negative	0	Negative
Negative	**UKBTB MN687**	0	Negative	0	Negative
Negative	**UKBTB MN688**	0	Negative	0	Negative
Negative	**UKBTB MN689**	0	Negative	0	Negative
Negative	**UKBTB MN690**	3	Positive	1	Negative
Negative	**UKBTB MN691**	0	Negative	0	Negative
Negative	**UKBTB MN692**	0	Negative	0	Negative
Negative	**UKBTB MN693**	0	Negative	0	Negative
Negative	**UKBTB MN694**	0	Negative	0	Negative
Negative	**UKBTB MN695**	0	Negative	0	Negative
Negative	**UKBTB MN696**	0	Negative	0	Negative
Negative	**UKBTB MN697**	0	Negative	0	Negative
Negative	**UKBTB MN698**	0	Negative	0	Negative
Negative	**UKBTB MN699**	0	Negative	0	Negative
Negative	**UKBTB MN700**	0	Negative	0	Negative
Negative	**UKBTB MN701**	0	Negative	0	Negative
Negative	**UKBTB MN702**	0	Negative	0	Negative
Negative	**UKBTB MN703**	0	Negative	0	Negative
Negative	**UKBTB MN704**	0	Negative	0	Negative
Negative	**UKBTB MN705**	0	Negative	0	Negative

Negative	**UKBTB MN706**	0	Negative	0	Negative
Negative	**UKBTB MN707**	0	Negative	0	Negative
Negative	**UKBTB MN708**	0	Negative	0	Negative
Negative	**UKBTB MN709**	0	Negative	0	Negative
Negative	**UKBTB MN710**	0	Negative	0	Negative
Negative	**UKBTB MN711**	0	Negative	0	Negative
Negative	**UKBTB MN712**	0	Negative	0	Negative
Negative	**UKBTB MN713**	0	Negative	0	Negative
Negative	**UKBTB MN714**	0	Negative	0	Negative
Negative	**UKBTB MN715**	0	Negative	0	Negative
Negative	**UKBTB MN716**	0	Negative	0	Negative
Negative	**UKBTB MN717**	0	Negative	0	Negative
Negative	**UKBTB MN718**	0	Negative	0	Negative
Negative	**UKBTB MN719**	1	Negative	1	Negative
Negative	**UKBTB MN720**	0	Negative	0	Negative
Negative	**UKBTB MN721**	0	Negative	0	Negative
Negative	**UKBTB MN722**	0	Negative	0	Negative
Negative	**UKBTB MN723**	0	Negative	0	Negative
Negative	**UKBTB MN724**	0	Negative	0	Negative
Negative	**UKBTB MN725**	0	Negative	0	Negative
Negative	**UKBTB MN726**	0	Negative	0	Negative
Negative	**UKBTB MN727**	0	Negative	0	Negative
Negative	**UKBTB MN728**	0	Negative	0	Negative
Negative	**UKBTB MN729**	0	Negative	0	Negative
Negative	**UKBTB MN730**	0	Negative	0	Negative
Negative	**UKBTB MN731**	0	Negative	0	Negative
Negative	**UKBTB MN732**	0	Negative	0	Negative
Negative	**UKBTB MN733**	0	Negative	0	Negative
Negative	**UKBTB MN734**	0	Negative	0	Negative
Negative	**UKBTB MN735**	0	Negative	0	Negative
Negative	**UKBTB MN736**	0	Negative	0	Negative

Negative	**UKBTB MN737**	0	Negative	0	Negative
Negative	**UKBTB MN738**	0	Negative	0	Negative
Negative	**UKBTB MN739**	0	Negative	0	Negative
Negative	**UKBTB MN740**	0	Negative	0	Negative
Negative	**UKBTB MN741**	0	Negative	0	Negative
Negative	**UKBTB MN742**	0	Negative	0	Negative
Negative	**UKBTB MN743**	0	Negative	0	Negative
Negative	**UKBTB MN744**	0	Negative	0	Negative
Negative	**UKBTB MN745**	1	Negative	0	Negative
Negative	**UKBTB MN746**	0	Negative	0	Negative
Negative	**UKBTB MN747**	0	Negative	0	Negative
Negative	**UKBTB MN748**	0	Negative	0	Negative
Negative	**UKBTB MN749**	0	Negative	0	Negative
Negative	**UKBTB MN750**	0	Negative	0	Negative
Negative	**UKBTB MN751**	0	Negative	0	Negative
Negative	**UKBTB MN752**	0	Negative	0	Negative
Negative	**UKBTB MN753**	0	Negative	0	Negative
Negative	**UKBTB MN754**	0	Negative	0	Negative
Negative	**UKBTB MN755**	0	Negative	0	Negative
Negative	**UKBTB MN756**	0	Negative	0	Negative
Negative	**UKBTB MN757**	0	Negative	0	Negative
Negative	**UKBTB MN758**	0	Negative	0	Negative
Negative	**UKBTB MN759**	0	Negative	0	Negative
Negative	**UKBTB MN760**	0	Negative	0	Negative
Negative	**UKBTB MN761**	0	Negative	0	Negative
Negative	**UKBTB MN762**	0	Negative	0	Negative
Negative	**UKBTB MN763**	0	Negative	0	Negative
Negative	**UKBTB MN764**	0	Negative	0	Negative
Negative	**UKBTB MN765**	0	Negative	0	Negative
Negative	**UKBTB MN766**	0	Negative	0	Negative
Negative	**UKBTB MN767**	0	Negative	0	Negative

Negative	**UKBTB MN768**	0	Negative	0	Negative
Negative	**UKBTB MN769**	0	Negative	0	Negative
Negative	**UKBTB MN770**	0	Negative	0	Negative
Negative	**UKBTB MN771**	0	Negative	0	Negative
Negative	**UKBTB MN772**	0	Negative	0	Negative
Negative	**UKBTB MN773**	0	Negative	0	Negative
Negative	**UKBTB MN774**	0	Negative	0	Negative
Negative	**UKBTB MN775**	0	Negative	0	Negative
Negative	**UKBTB MN776**	0	Negative	0	Negative
Negative	**UKBTB MN777**	0	Negative	0	Negative
Negative	**UKBTB MN778**	0	Negative	0	Negative
Negative	**UKBTB MN779**	0	Negative	0	Negative
Negative	**UKBTB MN780**	0	Negative	0	Negative
Negative	**UKBTB MN781**	0	Negative	0	Negative
Negative	**UKBTB MN782**	0	Negative	0	Negative
Negative	**UKBTB MN783**	0	Negative	0	Negative
Negative	**UKBTB MN784**	0	Negative	0	Negative
Negative	**UKBTB MN785**	0	Negative	0	Negative
Negative	**UKBTB MN786**	0	Negative	0	Negative
Negative	**UKBTB MN787**	0	Negative	0	Negative
Negative	**UKBTB MN788**	0	Negative	0	Negative
Negative	**UKBTB MN789**	0	Negative	0	Negative
Negative	**UKBTB MN790**	0	Negative	0	Negative
Negative	**UKBTB MN791**	0	Negative	0	Negative
Negative	**UKBTB MN792**	0	Negative	0	Negative
Negative	**UKBTB MN793**	0	Negative	0	Negative
Negative	**UKBTB MN794**	0	Negative	0	Negative
Negative	**UKBTB MN795**	0	Negative	0	Negative
Negative	**UKBTB MN796**	0	Negative	0	Negative
Negative	**UKBTB MN797**	0	Negative	0	Negative
Negative	**UKBTB MN798**	0	Negative	0	Negative

Negative	**UKBTB MN799**	0	Negative	0	Negative
Negative	**UKBTB MN800**	0	Negative	0	Negative
Negative	**UKBTB MN801**	0	Negative	0	Negative
Negative	**UKBTB MN802**	0	Negative	0	Negative
Negative	**UKBTB MN803**	0	Negative	0	Negative
Negative	**UKBTB MN804**	0	Negative	0	Negative
Negative	**UKBTB MN805**	0	Negative	0	Negative
Negative	**UKBTB MN806**	0	Negative	0	Negative
Negative	**UKBTB MN807**	0	Negative	0	Negative
Negative	**UKBTB MN808**	0	Negative	0	Negative
Negative	**UKBTB MN809**	0	Negative	0	Negative
Negative	**UKBTB MN810**	0	Negative	0	Negative
Negative	**UKBTB MN811**	0	Negative	0	Negative
Negative	**UKBTB MN812**	0	Negative	0	Negative
Negative	**UKBTB MN813**	0	Negative	0	Negative
Negative	**UKBTB MN814**	0	Negative	0	Negative
Negative	**UKBTB MN815**	0	Negative	0	Negative
Negative	**UKBTB MN816**	0	Negative	0	Negative
Negative	**UKBTB MN817**	0	Negative	0	Negative
Negative	**UKBTB MN818**	0	Negative	0	Negative
Negative	**UKBTB MN819**	0	Negative	0	Negative
Negative	**UKBTB MN820**	0	Negative	0	Negative
Negative	**UKBTB MN821**	0	Negative	0	Negative
Negative	**UKBTB MN822**	0	Negative	0	Negative
Negative	**UKBTB MN823**	0	Negative	0	Negative
Negative	**UKBTB MN824**	0	Negative	0	Negative
Negative	**UKBTB MN825**	0	Negative	0	Negative
Negative	**UKBTB MN826**	0	Negative	0	Negative
Negative	**UKBTB MN827**	0	Negative	0	Negative
Negative	**UKBTB MN828**	0	Negative	0	Negative
Negative	**UKBTB MN829**	0	Negative	0	Negative

Negative	**UKBTB MN830**	0	Negative	0	Negative
Negative	**UKBTB MN831**	0	Negative	0	Negative
Negative	**UKBTB MN832**	0	Negative	0	Negative
Negative	**UKBTB MN833**	0	Negative	0	Negative
Negative	**UKBTB MN834**	0	Negative	0	Negative
Negative	**UKBTB MN835**	0	Negative	0	Negative
Negative	**UKBTB MN836**	0	Negative	0	Negative
Negative	**UKBTB MN837**	0	Negative	0	Negative
Negative	**UKBTB MN838**	1	Negative	1	Negative
Negative	**UKBTB MN839**	0	Negative	0	Negative
Negative	**UKBTB MN840**	0	Negative	0	Negative
Negative	**UKBTB MN841**	0	Negative	0	Negative
Negative	**UKBTB MN842**	0	Negative	0	Negative
Negative	**UKBTB MN843**	0	Negative	0	Negative
Negative	**UKBTB MN844**	0	Negative	0	Negative
Negative	**UKBTB MN847**	1	Negative	0	Negative
Negative	**UKBTB MN848**	0	Negative	0	Negative
Negative	**UKBTB MN849**	0	Negative	0	Negative
Negative	**UKBTB MN850**	0	Negative	0	Negative
Negative	**UKBTB MN851**	0	Negative	0	Negative
Negative	**UKBTB MN852**	0	Negative	0	Negative
Negative	**UKBTB MN853**	0	Negative	0	Negative
Negative	**UKBTB MN854**	0	Negative	0	Negative
Negative	**UKBTB MN855**	0	Negative	0	Negative
Negative	**UKBTB MN856**	0	Negative	0	Negative
Negative	**UKBTB MN907**	0	Negative	0	Negative
Negative	**UKBTB MN908**	0	Negative	0	Negative
Negative	**UKBTB MN909**	0	Negative	0	Negative
Negative	**UKBTB MN910**	0	Negative	0	Negative
Negative	**UKBTB MN911**	0	Negative	0	Negative
Negative	**UKBTB MN912**	0	Negative	0	Negative

Negative	**UKBTB MN913**	0	Negative	0	Negative
Negative	**UKBTB MN914**	0	Negative	0	Negative
Negative	**UKBTB MN915**	0	Negative	0	Negative
Negative	**UKBTB MN916**	0	Negative	0	Negative
Negative	**UKBTB MN917**	0	Negative	0	Negative
Negative	**UKBTB MN918**	0	Negative	0	Negative
Negative	**UKBTB MN919**	0	Negative	0	Negative
Negative	**UKBTB MN920**	0	Negative	0	Negative
Negative	**UKBTB MN921**	0	Negative	0	Negative
Negative	**UKBTB MN922**	0	Negative	0	Negative
Negative	**UKBTB MN923**	0	Negative	0	Negative
Negative	**UKBTB MN924**	0	Negative	0	Negative
Negative	**UKBTB MN925**	0	Negative	0	Negative
Negative	**UKBTB MN926**	0	Negative	0	Negative
Negative	**UKBTB MN927**	0	Negative	0	Negative
Negative	**UKBTB MN928**	0	Negative	0	Negative
Negative	**UKBTB MN929**	0	Negative	0	Negative
Negative	**UKBTB MN930**	0	Negative	0	Negative
Negative	**UKBTB MN931**	0	Negative	0	Negative
Negative	**UKBTB MN932**	0	Negative	0	Negative
Negative	**UKBTB MN933**	0	Negative	0	Negative
Negative	**UKBTB MN934**	0	Negative	0	Negative
Negative	**UKBTB MN935**	0	Negative	0	Negative
Negative	**UKBTB MN936**	0	Negative	0	Negative
Negative	**UKBTB MN937**	0	Negative	0	Negative
Negative	**UKBTB MN938**	0	Negative	0	Negative
Negative	**UKBTB MN939**	0	Negative	0	Negative
Negative	**UKBTB MN940**	0	Negative	0	Negative
Negative	**UKBTB MN941**	0	Negative	0	Negative
Negative	**UKBTB MN942**	0	Negative	0	Negative
Negative	**UKBTB MN943**	0	Negative	0	Negative

Negative	**UKBTB MN944**	1	Negative	0	Negative
Negative	**UKBTB MN945**	0	Negative	0	Negative
Negative	**UKBTB MN946**	0	Negative	0	Negative
Negative	**UKBTB MN947**	0	Negative	0	Negative
Negative	**UKBTB MN948**	0	Negative	0	Negative
Negative	**UKBTB MN949**	1	Negative	1	Negative
Negative	**UKBTB MN950**	0	Negative	0	Negative
Negative	**UKBTB MN951**	0	Negative	0	Negative
Negative	**UKBTB MN952**	0	Negative	0	Negative
Negative	**UKBTB MN953**	0	Negative	0	Negative
Negative	**UKBTB MN954**	0	Negative	0	Negative
Negative	**UKBTB MN955**	0	Negative	0	Negative
Negative	**UKBTB MN956**	0	Negative	0	Negative
Negative	**UKBTB MN957**	0	Negative	0	Negative
Negative	**UKBTB MN958**	0	Negative	0	Negative
Negative	**UKBTB MN959**	0	Negative	0	Negative
Negative	**UKBTB MN960**	0	Negative	0	Negative
Negative	**UKBTB MN961**	0	Negative	0	Negative
Negative	**UKBTB MN962**	0	Negative	0	Negative
Negative	**UKBTB MN963**	0	Negative	0	Negative
Negative	**UKBTB MN964**	0	Negative	0	Negative
Negative	**UKBTB MN965**	0	Negative	0	Negative
Negative	**UKBTB MN966**	0	Negative	0	Negative
Negative	**UKBTB MN967**	0	Negative	0	Negative
Negative	**UKBTB MN968**	0	Negative	0	Negative
Negative	**UKBTB MN969**	0	Negative	0	Negative
Negative	**UKBTB MN970**	0	Negative	0	Negative
Negative	**UKBTB MN971**	1	Negative	0	Negative
Negative	**UKBTB MN972**	0	Negative	0	Negative
Negative	**UKBTB MN973**	0	Negative	0	Negative
Negative	**UKBTB MN974**	0	Negative	0	Negative

Negative	**UKBTB MN975**	0	Negative	0	Negative
Negative	**UKBTB MN976**	0	Negative	0	Negative
Negative	**UKBTB MN977**	0	Negative	0	Negative
Negative	**UKBTB MN978**	0	Negative	0	Negative
Negative	**UKBTB MN979**	0	Negative	0	Negative
Negative	**UKBTB MN980**	0	Negative	0	Negative
Negative	**UKBTB MN981**	0	Negative	0	Negative
Negative	**UKBTB MN982**	0	Negative	0	Negative
Negative	**UKBTB MN983**	0	Negative	0	Negative
Negative	**UKBTB MN984**	0	Negative	0	Negative
Negative	**UKBTB MN985**	0	Negative	0	Negative
Negative	**UKBTB MN986**	0	Negative	0	Negative
Negative	**UKBTB MN987**	1	Negative	0	Negative
Negative	**UKBTB MN988**	0	Negative	0	Negative
Negative	**UKBTB MN989**	0	Negative	0	Negative
Negative	**UKBTB MN990**	0	Negative	0	Negative
Negative	**UKBTB MN991**	0	Negative	0	Negative
Negative	**UKBTB MN992**	0	Negative	0	Negative
Negative	**UKBTB MN994**	0	Negative	0	Negative
Negative	**UKBTB MN995**	0	Negative	0	Negative
Negative	**UKBTB MN996**	0	Negative	0	Negative
Negative	**UKBTB MN997**	0	Negative	0	Negative
Negative	**UKBTB MN998**	0	Negative	0	Negative
Negative	**UKBTB MN999**	0	Negative	0	Negative
Negative	**UKBTB MN1000**	0	Negative	0	Negative
Negative	**UKBTB MN1001**	0	Negative	0	Negative
Negative	**UKBTB MN1002**	0	Negative	0	Negative
Negative	**UKBTB MN1003**	0	Negative	0	Negative
Negative	**UKBTB MN1004**	0	Negative	0	Negative
Negative	**UKBTB MN1005**	0	Negative	0	Negative
Negative	**UKBTB MN1006**	0	Negative	0	Negative

Negative	**UKBTB MN1007**	0	Negative	0	Negative
Negative	**UKBTB MN1008**	0	Negative	0	Negative
Negative	**UKBTB MN1009**	0	Negative	0	Negative
Negative	**UKBTB MN1010**	1	Negative	1	Negative
Negative	**UKBTB MN1011**	0	Negative	0	Negative
Negative	**UKBTB MN1012**	0	Negative	0	Negative
Negative	**UKBTB MN1013**	0	Negative	0	Negative
Negative	**UKBTB MN1014**	0	Negative	0	Negative
Negative	**UKBTB MN1015**	0	Negative	0	Negative
Negative	**UKBTB MN1016**	0	Negative	0	Negative
Negative	**UKBTB MN1017**	0	Negative	0	Negative
Negative	**UKBTB MN1018**	0	Negative	0	Negative
Negative	**UKBTB MN1019**	0	Negative	0	Negative
Negative	**UKBTB MN1020**	0	Negative	0	Negative
Negative	**UKBTB MN1021**	0	Negative	0	Negative
Negative	**UKBTB MN1022**	0	Negative	0	Negative
Negative	**UKBTB MN1023**	0	Negative	0	Negative
Negative	**UKBTB MN1024**	0	Negative	0	Negative
Negative	**UKBTB MN1025**	0	Negative	0	Negative
Negative	**UKBTB MN1026**	1	Negative	0	Negative
Negative	**UKBTB MN1027**	0	Negative	0	Negative
Negative	**UKBTB MN1028**	0	Negative	0	Negative
Negative	**UKBTB MN1029**	0	Negative	0	Negative
Negative	**UKBTB MN1030**	0	Negative	0	Negative
Negative	**UKBTB MN1031**	0	Negative	0	Negative
Negative	**UKBTB MN1032**	0	Negative	0	Negative
Negative	**UKBTB MN1033**	0	Negative	0	Negative
Negative	**UKBTB MN1034**	1	Negative	0	Negative
Negative	**UKBTB MN1035**	0	Negative	0	Negative
Negative	**UKBTB MN1036**	0	Negative	0	Negative
Negative	**UKBTB MN1037**	0	Negative	0	Negative

Negative	**UKBTB MN1038**	0	Negative	0	Negative
Negative	**UKBTB MN1039**	0	Negative	0	Negative
Negative	**UKBTB MN1040**	0	Negative	0	Negative
Negative	**UKBTB MN1041**	0	Negative	0	Negative
Negative	**UKBTB MN1042**	0	Negative	0	Negative
Negative	**UKBTB MN1043**	0	Negative	0	Negative
Negative	**UKBTB MN1044**	1	Negative	0	Negative
Negative	**UKBTB MN1045**	0	Negative	0	Negative
Negative	**UKBTB MN1046**	0	Negative	0	Negative
Negative	**UKBTB MN1047**	0	Negative	0	Negative
Negative	**UKBTB MN1048**	0	Negative	0	Negative
Negative	**UKBTB MN1049**	0	Negative	0	Negative
Negative	**UKBTB MN1050**	0	Negative	0	Negative
Negative	**UKBTB MN1051**	0	Negative	0	Negative
Negative	**UKBTB MN1052**	0	Negative	0	Negative
Negative	**UKBTB MN1053**	0	Negative	0	Negative
Negative	**UKBTB MN1054**	0	Negative	0	Negative
Negative	**UKBTB MN1055**	0	Negative	0	Negative
Negative	**UKBTB MN1056**	0	Negative	0	Negative
Negative	**UKBTB MN1057**	0	Negative	0	Negative
Negative	**UKBTB MN1058**	0	Negative	0	Negative
Negative	**UKBTB MN1059**	0	Negative	0	Negative
Negative	**UKBTB MN1060**	4	Positive	4	Positive
Negative	**UKBTB MN1061**	0	Negative	0	Negative
Negative	**UKBTB MN1062**	0	Negative	0	Negative
Negative	**UKBTB MN1063**	0	Negative	0	Negative
Negative	**UKBTB MN1064**	0	Negative	0	Negative
Negative	**UKBTB MN1065**	0	Negative	0	Negative
Negative	**UKBTB MN1066**	0	Negative	0	Negative
Negative	**UKBTB MN1067**	0	Negative	0	Negative
Negative	**UKBTB MN1068**	0	Negative	0	Negative

Negative	**UKBTB MN1069**	0	Negative	0	Negative
Negative	**UKBTB MN1070**	0	Negative	0	Negative
Negative	**UKBTB MN1071**	0	Negative	0	Negative
Negative	**UKBTB MN1072**	0	Negative	0	Negative
Negative	**UKBTB MN1073**	0	Negative	0	Negative
Negative	**UKBTB MN1074**	0	Negative	0	Negative
Negative	**UKBTB MN1075**	0	Negative	0	Negative
Negative	**UKBTB MN1076**	0	Negative	0	Negative
Negative	**UKBTB MN1077**	0	Negative	0	Negative
Negative	**UKBTB MN1078**	0	Negative	0	Negative
Negative	**UKBTB MN1079**	0	Negative	0	Negative
Negative	**UKBTB MN1080**	0	Negative	0	Negative
Negative	**UKBTB MN1081**	0	Negative	0	Negative
Negative	**UKBTB MN1082**	0	Negative	0	Negative
Negative	**UKBTB MN1083**	0	Negative	0	Negative
Negative	**UKBTB MN1084**	0	Negative	0	Negative
Negative	**UKBTB MN1085**	0	Negative	0	Negative
Negative	**UKBTB MN1086**	0	Negative	0	Negative
Negative	**UKBTB MN1087**	0	Negative	0	Negative
Negative	**UKBTB MN1088**	0	Negative	0	Negative
Negative	**UKBTB MN1089**	0	Negative	0	Negative
Negative	**UKBTB MN1090**	0	Negative	0	Negative
Negative	**UKBTB MN1091**	0	Negative	0	Negative
Negative	**UKBTB MN1092**	0	Negative	0	Negative
Negative	**UKBTB MN1093**	0	Negative	0	Negative
Negative	**UKBTB MN1094**	0	Negative	0	Negative
Negative	**UKBTB MN1095**	0	Negative	0	Negative
Negative	**UKBTB MN1096**	0	Negative	0	Negative
Negative	**UKBTB MN1097**	0	Negative	0	Negative
Negative	**UKBTB MN1098**	0	Negative	0	Negative
Negative	**UKBTB MN1099**	0	Negative	0	Negative

Negative	**UKBTB MN1100**	0	Negative	0	Negative
Negative	**UKBTB MN1101**	1	Negative	0	Negative
Negative	**UKBTB MN1102**	0	Negative	0	Negative
Negative	**UKBTB MN1103**	0	Negative	0	Negative
Negative	**UKBTB MN1104**	0	Negative	0	Negative
Negative	**UKBTB MN1105**	0	Negative	0	Negative
Negative	**UKBTB MN1106**	0	Negative	0	Negative
Negative	**UKBTB MN1107**	0	Negative	0	Negative
Negative	**UKBTB MN1108**	0	Negative	0	Negative
Negative	**UKBTB MN1109**	0	Negative	0	Negative
Negative	**UKBTB MN1110**	0	Negative	0	Negative
Negative	**UKBTB MN1111**	0	Negative	0	Negative
Negative	**UKBTB MN1112**	0	Negative	0	Negative
Negative	**UKBTB MN1113**	0	Negative	0	Negative
Negative	**UKBTB MN1114**	0	Negative	0	Negative
Negative	**UKBTB MN1115**	0	Negative	0	Negative
Negative	**UKBTB MN1116**	0	Negative	0	Negative
Negative	**UKBTB MN1117**	0	Negative	0	Negative
Negative	**UKBTB MN1118**	0	Negative	0	Negative
Negative	**UKBTB MN1119**	0	Negative	0	Negative
Negative	**UKBTB MN1120**	0	Negative	0	Negative
Negative	**UKBTB MN1121**	0	Negative	0	Negative
Negative	**UKBTB MN1122**	0	Negative	0	Negative
Negative	**UKBTB MN1123**	0	Negative	0	Negative
Negative	**UKBTB MN1124**	0	Negative	0	Negative
Negative	**UKBTB MN1125**	0	Negative	0	Negative
Negative	**UKBTB MN1126**	0	Negative	0	Negative
Negative	**UKBTB MN1127**	0	Negative	0	Negative
Negative	**UKBTB MN1128**	0	Negative	0	Negative
Negative	**UKBTB MN1129**	0	Negative	0	Negative
Negative	**UKBTB MN1130**	0	Negative	0	Negative

Negative	**UKBTB MN1131**	0	Negative	0	Negative
Negative	**UKBTB MN1132**	0	Negative	0	Negative
Negative	**UKBTB MN1133**	0	Negative	0	Negative
Negative	**UKBTB MN1134**	0	Negative	0	Negative
Negative	**UKBTB MN1135**	0	Negative	0	Negative
Negative	**UKBTB MN1136**	0	Negative	0	Negative
Negative	**UKBTB MN1137**	0	Negative	0	Negative
Negative	**UKBTB MN1138**	0	Negative	0	Negative
Negative	**UKBTB MN1139**	0	Negative	0	Negative
Negative	**UKBTB MN1140**	0	Negative	0	Negative
Negative	**UKBTB MN1141**	0	Negative	0	Negative
Negative	**UKBTB MN1142**	0	Negative	0	Negative
Negative	**UKBTB MN1143**	0	Negative	0	Negative
Negative	**UKBTB MN1144**	0	Negative	0	Negative
Negative	**UKBTB MN1145**	0	Negative	0	Negative
Negative	**UKBTB MN1146**	0	Negative	0	Negative
Negative	**UKBTB MN1147**	0	Negative	0	Negative
Negative	**UKBTB MN1148**	0	Negative	0	Negative
Negative	**UKBTB MN1149**	0	Negative	0	Negative
Negative	**UKBTB MN1150**	0	Negative	0	Negative
Negative	**UKBTB MN1151**	0	Negative	0	Negative
Negative	**UKBTB MN1152**	0	Negative	0	Negative
Negative	**UKBTB MN1153**	0	Negative	0	Negative
Negative	**UKBTB MN1154**	1	Negative	0	Negative
Negative	**UKBTB MN1155**	0	Negative	0	Negative
Negative	**UKBTB MN1156**	0	Negative	0	Negative
Negative	**UKBTB MN1157**	0	Negative	0	Negative
Negative	**UKBTB MN1158**	0	Negative	0	Negative
Negative	**UKBTB MN1159**	0	Negative	0	Negative
Negative	**UKBTB MN1160**	0	Negative	0	Negative
Negative	**UKBTB MN1161**	0	Negative	0	Negative

Negative	**UKBTB MN1162**	0	Negative	0	Negative
Negative	**UKBTB MN1163**	0	Negative	0	Negative
Negative	**UKBTB MN1164**	0	Negative	0	Negative
Negative	**UKBTB MN1165**	4	Positive	3	Positive
Negative	**UKBTB MN1166**	0	Negative	0	Negative
Negative	**UKBTB MN1167**	0	Negative	0	Negative
Negative	**UKBTB MN1168**	0	Negative	0	Negative
Negative	**UKBTB MN1169**	0	Negative	0	Negative
Negative	**UKBTB MN1170**	0	Negative	0	Negative
Negative	**UKBTB MN1171**	0	Negative	0	Negative
Negative	**UKBTB MN1172**	0	Negative	0	Negative
Negative	**UKBTB MN1173**	0	Negative	0	Negative
Negative	**UKBTB MN1174**	0	Negative	0	Negative
Negative	**UKBTB MN1175**	1	Negative	0	Negative
Negative	**UKBTB MN1176**	0	Negative	0	Negative
Negative	**UKBTB MN1177**	0	Negative	0	Negative
Negative	**UKBTB MN1178**	0	Negative	0	Negative
Negative	**UKBTB MN1179**	0	Negative	0	Negative
Negative	**UKBTB MN1180**	0	Negative	0	Negative
Negative	**UKBTB MN1181**	0	Negative	0	Negative
Negative	**UKBTB MN1182**	0	Negative	0	Negative
Negative	**UKBTB MN1183**	0	Negative	0	Negative
Negative	**UKBTB MN1184**	0	Negative	0	Negative
Negative	**UKBTB MN1185**	0	Negative	0	Negative
Negative	**UKBTB MN1186**	0	Negative	0	Negative
Negative	**UKBTB MN1187**	0	Negative	0	Negative
Negative	**UKBTB MN1188**	0	Negative	0	Negative
Negative	**UKBTB MN1189**	0	Negative	0	Negative
Negative	**UKBTB MN1190**	0	Negative	0	Negative
Negative	**UKBTB MN1191**	0	Negative	0	Negative
Negative	**UKBTB MN1192**	0	Negative	0	Negative

Negative	**UKBTB MN1193**	0	Negative	0	Negative
Negative	**UKBTB MN1194**	0	Negative	0	Negative
Negative	**UKBTB MN1195**	0	Negative	0	Negative
Negative	**UKBTB MN1196**	0	Negative	0	Negative
Negative	**UKBTB MN1197**	0	Negative	0	Negative
Negative	**UKBTB MN1198**	0	Negative	0	Negative
Negative	**UKBTB MN1199**	0	Negative	0	Negative
Negative	**UKBTB MN1200**	0	Negative	0	Negative
Negative	**UKBTB MN1201**	0	Negative	0	Negative
Negative	**UKBTB MN1202**	0	Negative	0	Negative
Negative	**UKBTB MN1203**	0	Negative	0	Negative
