# Supplementary material for: Diagnostic accuracy of the Enferplex Bovine TB antibody test using individual milk samples from cattle
Source: PLoS One. 2024 Apr 30;19(4):e0301609. doi: 10.1371/journal.pone.0301609 (PMC11060599; doi:10.1371/journal.pone.0301609)
Supplement: S1 Fig — The relative light unit (RLU) obtained with the blank spot was subtracted from the RLU value obtained from antigen spots to obtain a blanked RLU value for each serum and milk sample. Results obtained from serum are shown on the Y axis and from the milk samples are shown on the X axis. Spearman’s rank correlation coefficient ρ obtained with paired serum and milk samples for each antigen ranged between 0.779–0.955 across the 11 antigens (P <0.0001 for all antigens). (PDF) [file pone.0301609.s002.pdf]

S1 Fig. Correlation between serum and milk test results for 11 antigens. Results are expressed as relative light units (RLU).

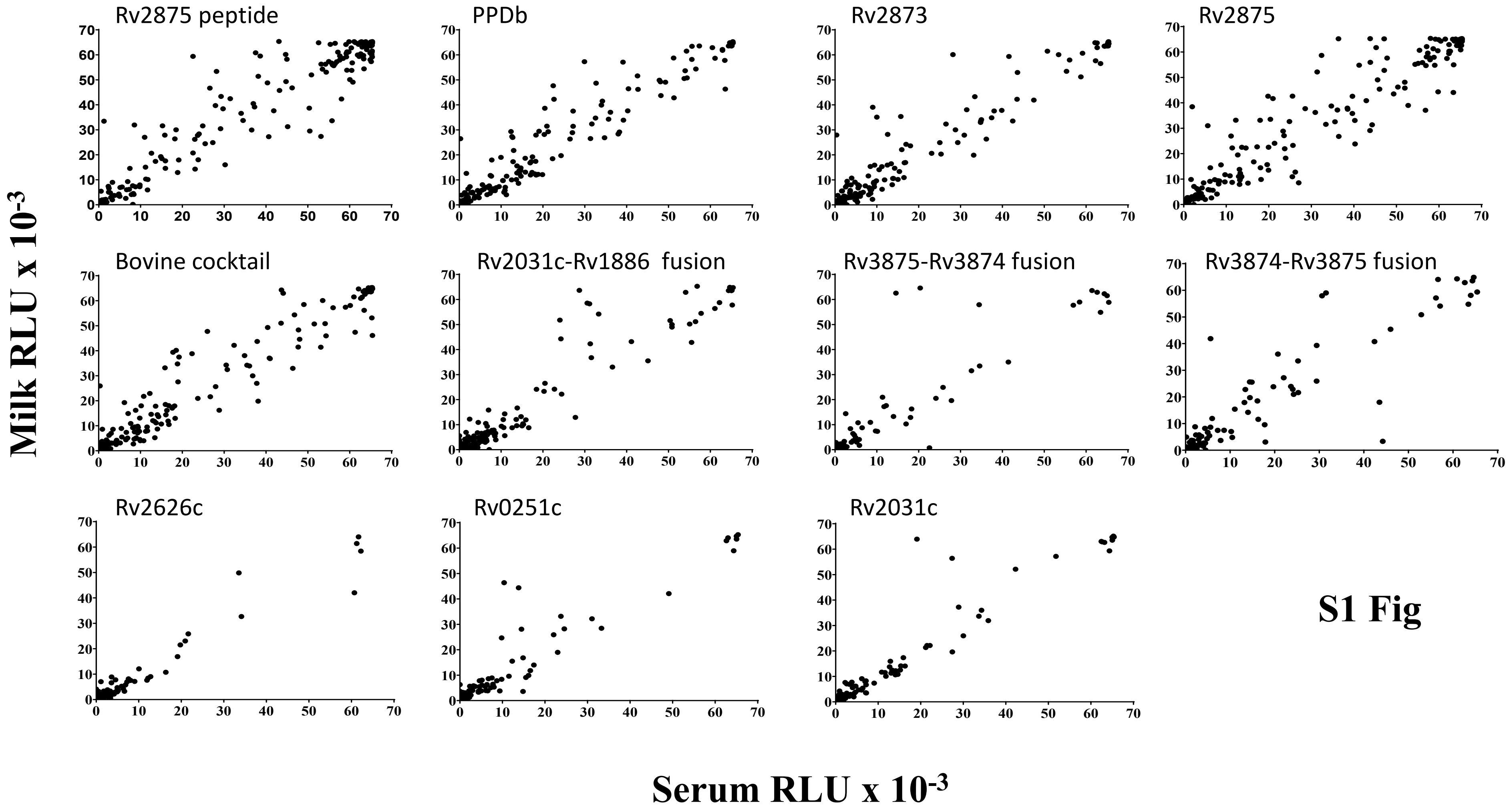

The relative light unit (RLU) obtained with the blank spot was subtracted from the RLU value obtained from antigen spots to obtain a blanked RLU value for each serum and milk sample. Results obtained from serum are shown on the Y axis and from the milk samples are shown on the X axis. Spearman's rank correlation coefficient ρ ranged from 0.756- 0.965, $P < 0.0001$.
