# Supplementary material for: Diagnostic accuracy of the Enferplex Bovine TB antibody test using individual milk samples from cattle
Source: PLoS One. 2024 Apr 30;19(4):e0301609. doi: 10.1371/journal.pone.0301609 (PMC11060599; doi:10.1371/journal.pone.0301609)

Plots of antigen 1 – 11 reproducibility obtained by 3 independent laboratories using individual milk – RLU and S/CO ratio data

An evaluation panel of individual milk samples comprising negative, weak positive and strong positive individual milk samples were blinded and sent to the 3 laboratories for analytical reproducibility testing. Seven negative samples, 7 weak positive samples, and 7 strong positive samples were tested in duplicate using two different kit batches and 1 technician in each laboratory. The results obtained for individual milk samples using raw RLU and S/CO ratio data are shown in the plots for each category of sample.

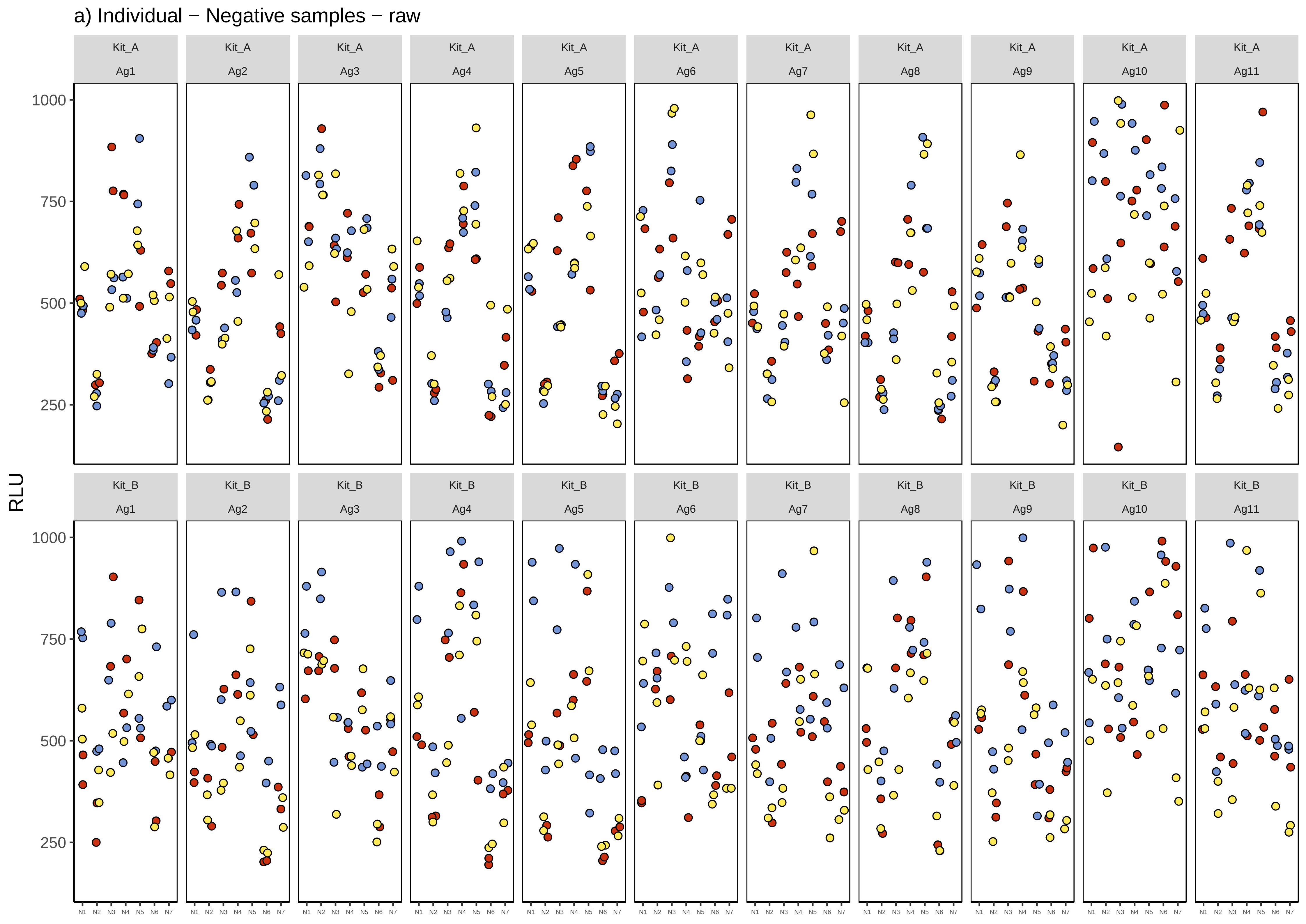
a) Individual − Negative samples − raw
RLU
Kit_A
Kit_B
Ag1
Ag2
Ag3
Ag4
Ag5
Ag6
Ag7
Ag8
Ag9
Ag10
Ag11
1000
750
500
250
N1
N2
N3
N4
N5
N6
N7

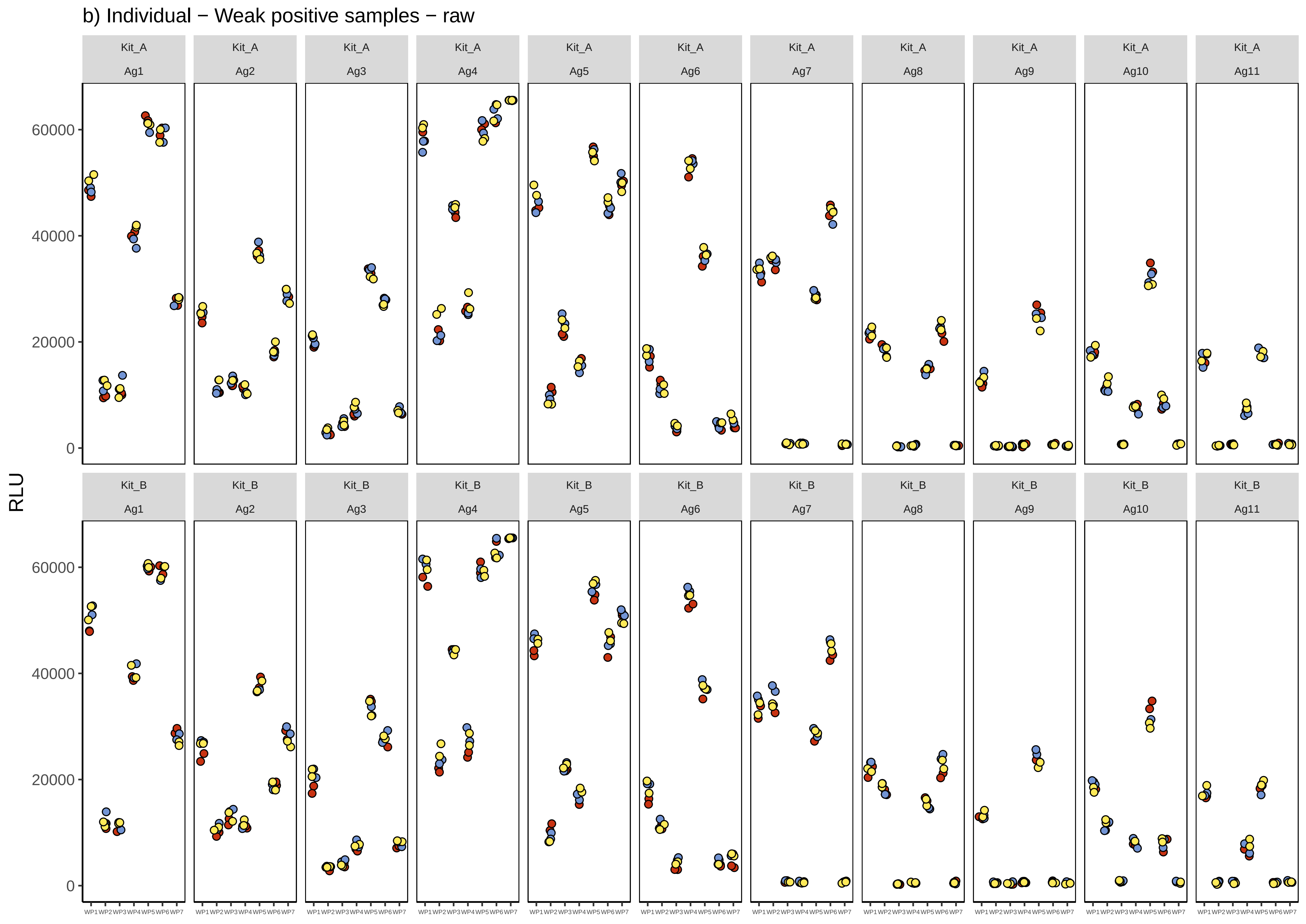
b) Individual − Weak positive samples − raw
Kit_A
Kit_B
Ag1
Ag2
Ag3
Ag4
Ag5
Ag6
Ag7
Ag8
Ag9
Ag10
Ag11
RLU
0
20000
40000
60000
WP1
WP2
WP3
WP4
WP5
WP6
WP7

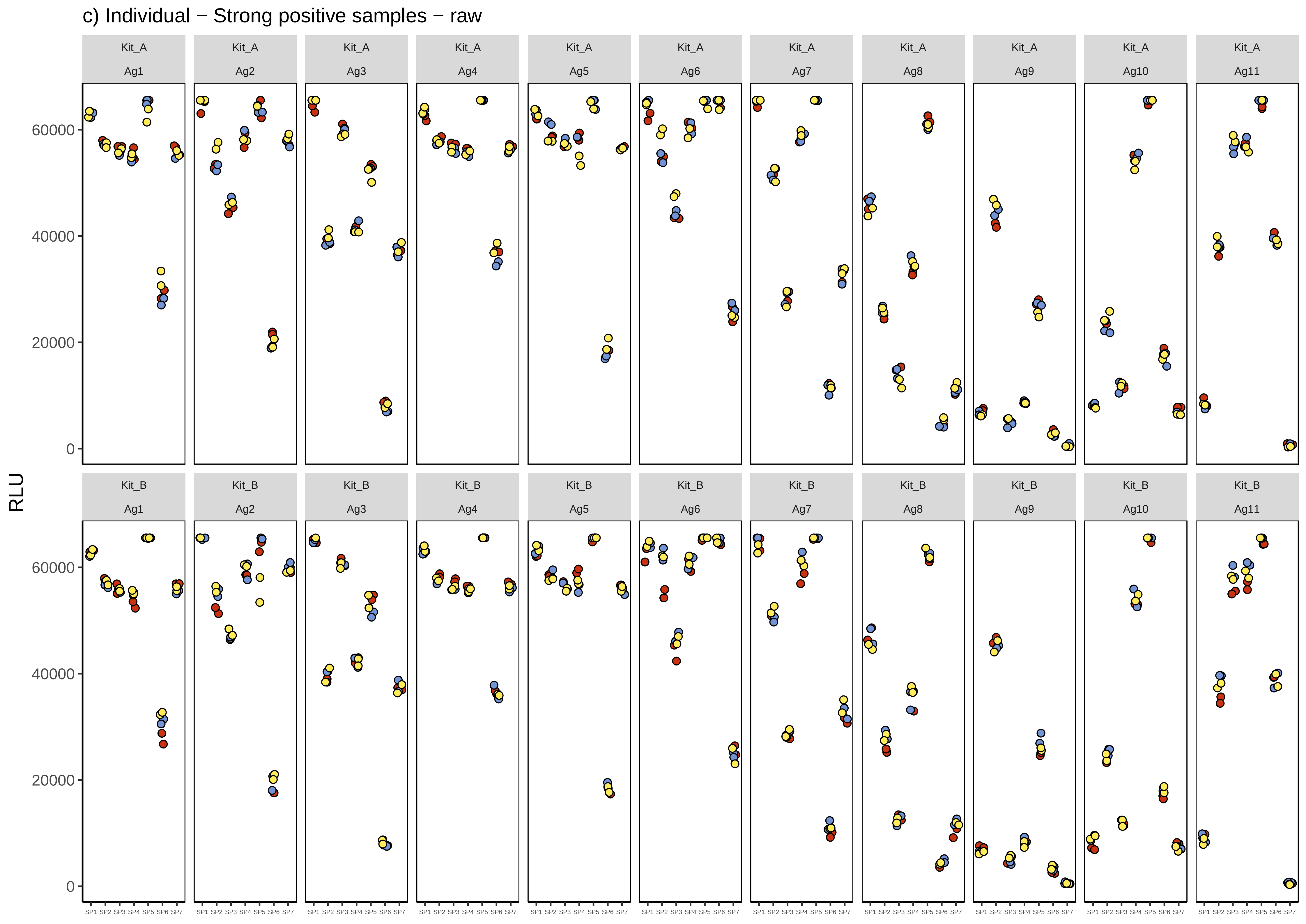

c) Individual − Strong positive samples − raw
Kit_A
Kit_B
Ag1
Ag2
Ag3
Ag4
Ag5
Ag6
Ag7
Ag8
Ag9
Ag10
Ag11
RLU
0
20000
40000
60000
SP1 SP2 SP3 SP4 SP5 SP6 SP7

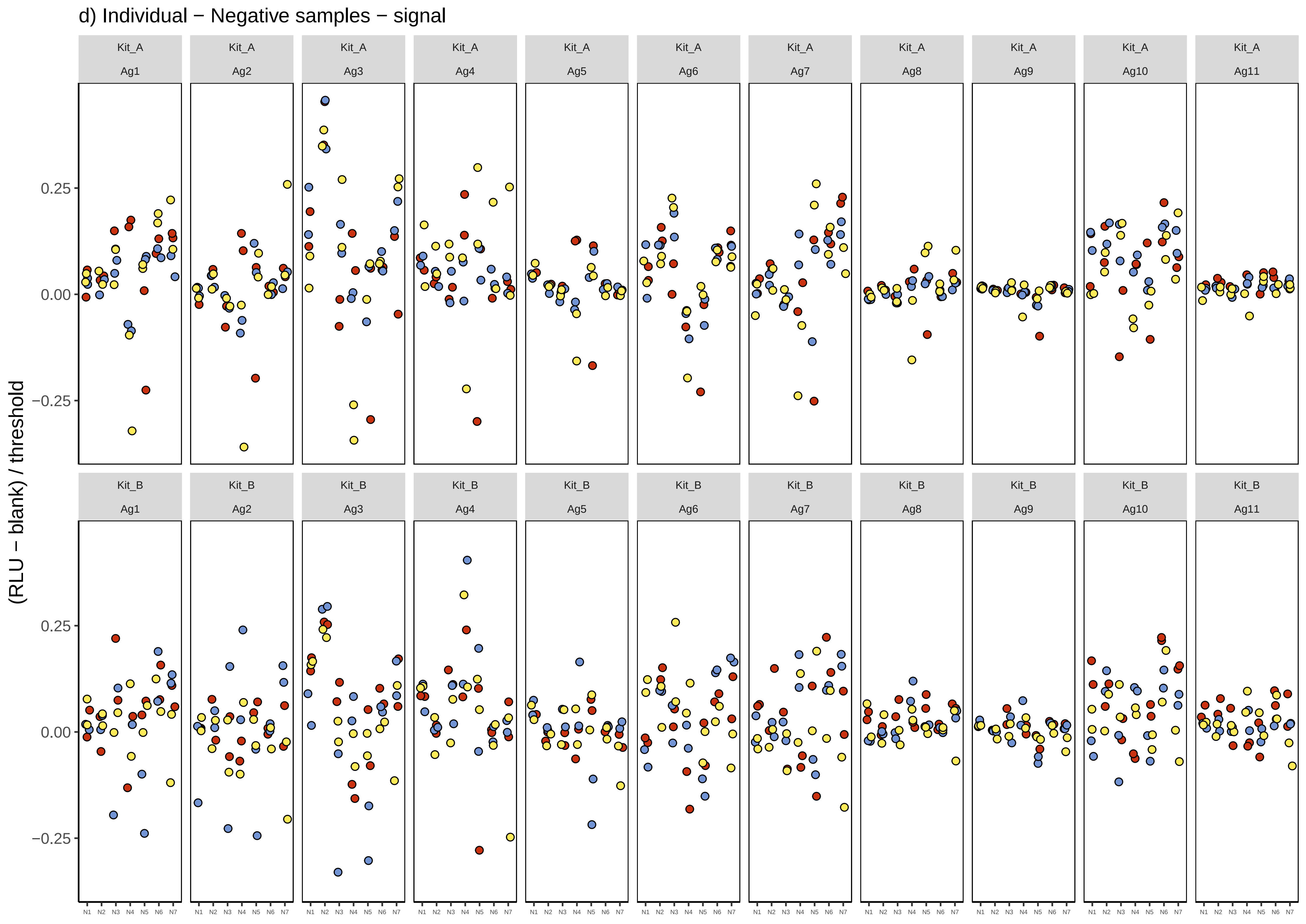

d) Individual − Negative samples − signal
(RLU − blank) / threshold
Kit_A
Kit_B
Ag1
Ag2
Ag3
Ag4
Ag5
Ag6
Ag7
Ag8
Ag9
Ag10
Ag11
0.25
0.00
−0.25
N1
N2
N3
N4
N5
N6
N7

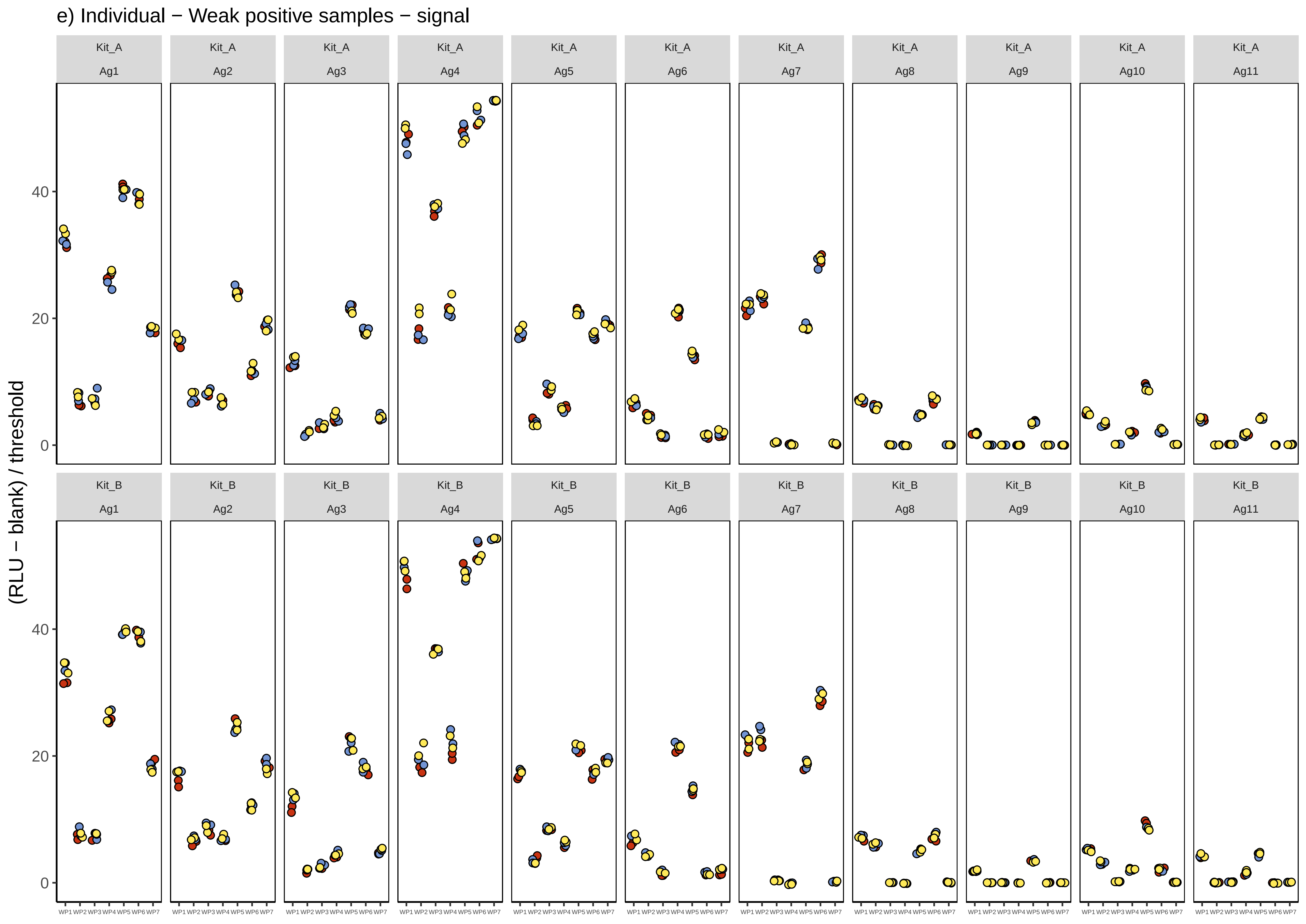
e) Individual − Weak positive samples − signal
Kit_A
Kit_B
Ag1
Ag2
Ag3
Ag4
Ag5
Ag6
Ag7
Ag8
Ag9
Ag10
Ag11
(RLU − blank) / threshold
0
20
40
WP1 WP2 WP3 WP4 WP5 WP6 WP7

f) Individual − Strong positive samples − signal

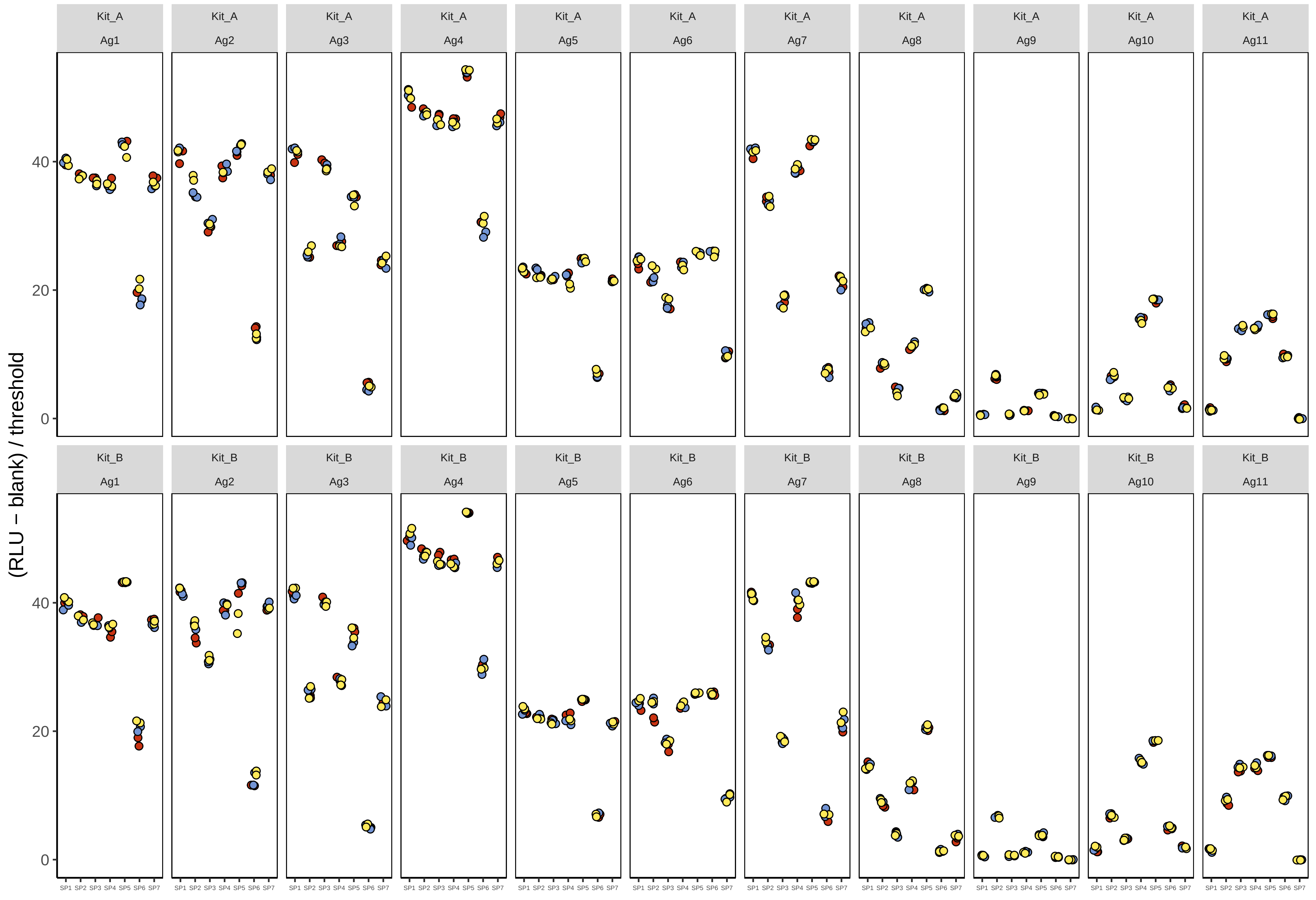

Supplement: S2 Fig — An evaluation panel of samples comprising negative, weak positive and strong positive milk samples were blinded and sent to three independent laboratories for reproducibility testing. Seven negative samples, 7 weak positive samples, and 7 strong positive samples (based on the two-antigen rule) were tested in duplicate using two plates from two different kit batches and one technician in each of the three independent laboratories. The results obtained for raw and signal/cut-off data are shown in the plots. (PDF) [file pone.0301609.s003.pdf]
